# Parental perceptions and attitudes towards the inclusion of children with neurodevelopmental, physical and sensory disabilities

**DOI:** 10.3389/fpsyt.2025.1735746

**Published:** 2026-01-20

**Authors:** Noémi Vivien Kárpáti, Mónika Miklósi

**Affiliations:** 1 Doctoral School of Psychology, ELTE, Eötvös Loránd University, Budapest, Hungary; 2 Institute of Psychology, Eötvös Loránd University, Budapest, Hungary; 3 Centre of Mental Health, Heim Pál National Pediatric Institute, Budapest, Hungary

**Keywords:** disability attitudes, helping self-efficacy, intergroup contact theory, network analysis, neurodevelopmental disorders, parental knowledge, perceived impact, social inclusion

## Abstract

**Objective:**

Parental knowledge and attitudes are key determinants of inclusive education and children’s social participation, yet little is known about how parents conceptualize different types of disabilities. This study explored patterns of perceived knowledge, ability to help, and attitudes toward structural and relational inclusion across multiple disability categories within a network-analytic framework.

**Method:**

A cross-sectional online survey was completed by 347 Hungarian parents. Respondents rated five domains (knowledge, perceived impact, ability to help, class inclusion, and friendship inclusion) and reported prior experiences for nine disability types (autism spectrum disorder, attention-deficit/hyperactivity disorder, anxiety disorders, intellectual disability, orthopedic impairment, visual impairment, hearing impairment, speech impairment, and Down syndrome). Network analyses (EBICglasso) were performed for six sufficiently represented conditions.

**Results:**

Overall, parents expressed favorable attitudes toward inclusion but reported limited self-efficacy in helping children with disabilities. Across networks, friendship and class inclusion formed a robust positive cluster, and perceived knowledge was positively linked to helping capability, although the strength of this connection varied across conditions. In contrast, perceived impact displayed negative edges with helping and inclusion willingness, particularly for ADHD, intellectual disability, and speech impairment.

**Conclusion:**

The findings highlight a consistent triadic structure connecting knowledge, ability to help, and inclusion willingness, with contact and perceived impact shaping their balance differently by disability type. Interventions promoting guided contact and targeted knowledge-building may enhance both perceived competence and inclusive attitudes. Future studies should adopt longitudinal or experimental designs, employ multi-item measures, and test causal pathways linking parental experience, knowledge, and inclusion behavior.

## 1 Introduction

Inclusion of people with disabilities has become a defining goal in contemporary social and health policy, reflecting a global shift toward participation, equity, and belonging (1, 2). For children, inclusion is not only a matter of educational access but a fundamental social and developmental need, as well as the right to full participation in public and cultural life, according to the UN Convention on the Rights of Persons with Disabilities (3). Therefore, it encompasses the ability to engage in everyday interactions, form peer relationships, and participate in community life without stigma or exclusion (4, 5). According to UNICEF (6), approximately one in ten children worldwide lives with a disability that may affect cognitive, physical, or socio-emotional functioning, yet barriers to participation remain widespread. In their review, Koller and colleagues (7) concluded that children with special educational needs often experience social exclusion in school settings, which impacts the quality of their social participation and overall well-being. A recent study conducted in Spain (8) directly involved typically and atypically developing children and found that the latter group reported significantly lower levels of social acceptance, well-being, and peer relations. Additionally, the study noted that 38.6% of students experienced bullying in school. Similarly, another study showed that disabled children perceive themselves to be at greater risk for bullying in school, and more than forty percent of them consider bullying to be normal (9). These experiences underscore that inclusion cannot be achieved solely by policy mandates or classroom integration; it requires attitudinal change and social acceptance driven by families and communities.

Social inclusion is more than physical presence—it involves meaningful participation, positive social interaction, and mutual acceptance within peer groups and communities. As Koster and colleagues (5) proposed, social participation represents the most complete form of inclusion, encompassing four dimensions: positive contact, peer acceptance, friendships, and the individual’s own sense of belonging. For children with disabilities, these elements are critical predictors of well-being, self-esteem, long-term social functioning (7, 8, 10), and quality of life (11, 12).

Research has established that providing specific and practical knowledge about mental health conditions in the form of psychoeducation could be an effective and relatively long-lasting solution for reducing negative attitudes, stigmatization, and improving the willingness and ability to help (13). It is, however, also stated that the public’s knowledge of some disabilities, such as intellectual disability, is generally low, due to a lack of awareness and confusion of terminology (14). This lack of understanding can sustain exclusionary attitudes and hinder the development of inclusive communities.

Parents have a significant influence on shaping inclusive environments. They are typically the first interpreters of their child’s difficulties and the primary gatekeepers to proper care. It has been demonstrated that higher parenting knowledge is positively associated with neurodevelopmental outcomes in cognitive, language, motor, and social-emotional domains (15). Bornstein and colleagues (16) assessed maternal knowledge of infant development using the *Knowledge of Infant Development Inventory* (KIDI) and found that, in a U.S. sample of 268 mothers, participants answered 81% of items correctly; knowledge increased with maternal age and education. On the other hand, evidence indicates that even in highly educated samples, factual understanding of disabilities such as specific learning disability was low; however, with a one-session psychoeducational intervention, knowledge of terminology, longevity of the disability, and treatment necessity was significantly increased (17). However, previous studies typically focused on a single disability [e.g. intellectual disability (14); Angelman Syndrome (18); specific learning disability (17)]; to our knowledge, no study investigated parents’ – including those of typically and atypically developing children – perceived knowledge on a broad range of disabilities simultaneously.

Studies have shown that the parental role in attitude formation and implementation of inclusion practices is significant (1, 2, 7). According to social learning theory, children internalize their parents’ attitudes and behaviors, reproducing them in social situations (19). Thus, parents’ openness toward diversity contributes directly to the development of inclusive peer cultures (20).

Often, parents of children with disabilities advocate for social inclusion and integration in education. They voice their concerns regarding risks of inclusion in mainstream school settings (4), but also have positive opinions and hopes about inclusion, such that their children are provided with more opportunities to contact typically developing children and to form friendships, potentially promoting acceptance from their peers (18). However, some evidence suggests that parents of typically developing children also recognize the potential benefits of inclusion, in terms of their children developing pro-social skills at an early age (2). Despite their importance, parental attitudes are still under-researched areas compared to those of teachers and students (1).

When researching general or parental attitudes, the potential impact of demographic characteristics, such as participants’ age, gender, residence, educational level, and overall socio-economic status, should always be considered. A review study (2) concluded that while neither the parents’ gender nor their age had a stable effect on their attitude towards inclusion, parents with a higher socio-economic status and higher education level reported more positive attitudes. Paseka and Schwab (1) found that for some disabilities (learning and mental disabilities), mothers seemed to be more accepting, while in other cases, there were no significant gender differences. Regarding the parents’ age, they also reported inconsistent results, where in some cases (behavioural disorders, mental disabilities) lower age meant more positive attitudes, while in others, no differences were detected. Additionally, parents of higher educational background and income reported more positive attitudes regarding the inclusion of children with physical disabilities, while, interestingly, those of lower educational levels had a more positive approach to children with learning disabilities (1).

Another important factor is prior experience, in our case, parents’ previous experiences with disabled children and their inclusion in education. De Boer and colleagues (2) found that more inclusion-related experience in education predicted more positive attitudes towards inclusion. Paseka and Schwab (1) also found that, although only a minority of parents were accepting of inclusion regardless of the type of disability, parents whose children attended inclusive classes were more supportive of including children with special educational needs. Another study (18) found that parents whose disabled children attended schools with inclusive classes reported more positive attitudes towards inclusive education than parents who were mostly familiar with segregated educational settings. Moreover, it is established that contact between children with disabilities and their typically developing peers helps reduce negative attitudes, highlighting that the quality of contact is of high importance, as negative experiences can have adverse effects (14, 21). Drawing on intergroup contact theory, prior experience with members of diverse ethnic or racial groups can be viewed as a key mechanism for reducing intergroup prejudice (22); the theory can be extended to other outgroups, such as individuals living with disabilities and mental health conditions. Recent evidence suggests that contact theory can be applied in the context of primary inclusive education, by demonstrating that the combination of personal contact and disability specific knowledge promoted the formation of positive peer attitudes, therefore improving the quality of social participation of students with disabilities (10).

There is evidence that the type of disability also has an impact on parents’ approach. One study (23) found that when comparing parents of children with and without disabilities, both groups reported the most positive attitudes towards children with physical and sensory disabilities, and were the least supportive of emotional problems and cognitive impairment, as well as autism spectrum disorder (ASD). Another study found that generally, people are more approaching to others with physical disabilities than intellectual disabilities, while severe mental health conditions are the least favorable in terms of social inclusion (14). Similarly, it was found that people hold more positive attitudes towards the inclusion of students with physical conditions, while behavioural problems and mental disabilities are the least accepted (1). Interestingly, in terms of social exclusion and bullying among students, another study (8) found no significant differences across different types of disabilities.

Hungary provides a particularly relevant case for studying these dynamics. In the 2024/2025 school year in Hungary, there was a reported total of 109430 children with special needs (24), about 8 percent of all preschool and school-age children (25). This includes 49558 (~3.6% of all children with special education needs) children with learning disability, 19626 (~1.4%) children with intellectual disability (mild or moderate), 13238 (~1%) children with ASD, 7875 (~0.6%) children with attention-deficit, 7072 (~0.5%) children with speech impairment, 3455 (~0.25%) children with conduct problems, 1762 (~0.12%) children with orthopedic impairment, 1599 (~0.11%) children with hearing impairment, 681 (~0.05%) children with visual impairment, and an additional 4564 (~0.3%) comorbid incidences. In 2020, 49 out of every 1000 children were born with a congenital disorder, and in 2022, there were 225 reported cases of children with Down syndrome (26). Despite legislative progress, many families still encounter systemic barriers to inclusion, from limited support services to inconsistent community acceptance (27).

Public attitudes largely mirror these structural challenges. According to a Hungarian representative social report (28), which investigated the general public’s attitudes towards children and adults living with one of five different disabilities (ASD, intellectual disability, orthopedic impairment, visual impairment, hearing impairment), it was found that overall, people reported rather positive attitudes; however, the results varied across different disabilities. Hungarian adults showed the least supportive attitudes towards ASD and intellectual disability, while they showed a high willingness to include people living with orthopedic and sensory impairment. This difference manifested in terms of opinions on marriage, child rearing, employment, voting rights, leisure activities, and, in the case of children, the inclusion in mainstream education. Interestingly, people were more supportive of children than adults with the same disability, when considering their rights to access to different cultural and leisure activities, suggesting that empathy and social acceptance are more readily extended to younger populations. These findings highlight the importance of parental perspectives in shaping everyday inclusion, both in structured settings and in informal community life.

While a substantial body of research focuses on social inclusion of children with disabilities from an educational point of view, the importance of everyday interactions and peer relations outside of institutional boundaries is often overlooked. Although inclusion has become a central concept in policy and research, relatively little is known about how parents simultaneously perceive different dimensions of disability and inclusion in both social and structural contexts. Most prior studies focused on education-specific settings or examined single aspects of inclusion, such as attitudes or knowledge, in isolation. The present study aims to bridge this gap by adopting a multidimensional approach to explore Hungarian parents’ perceptions and attitudes across five domains toward children with nine types of disabilities: ASD, attention-deficit/hyperactivity disorder (ADHD), anxiety disorders, intellectual disability, orthopedic impairment, visual impairment, hearing impairment, speech impairment, and Down syndrome.

We aimed to explore parental perceptions of each disability across the following domains: perceived knowledge, perceived impact on the child’s life, attitudes toward *structural inclusion* (participation in educational settings), attitudes toward *relational inclusion* (friendship and social interaction), and perceived ability to help affected children or families. In order to gain further insight into the relationships between these constructs and identify key factors that can be influenced to increase support for inclusion and the ability to help, network analysis was applied. By understanding how parents conceptualize and engage with disability, the research aims to inform strategies that foster more inclusive, equitable, and compassionate communities for all children.

## 2 Method

### 2.1 Sample and procedures

The Institutional Research Ethics Committee of the Psychological Institute of Eötvös Loránd University approved the study (Nr. 2024/408). We included a community sample of parents with at least one child aged 3 to 12 years. The call for participation was put out via the internet, through social media platforms, and in person by distributing fliers in various points of the capital and other cities. In all cases, after providing their informed consent, parents completed an online questionnaire package accessible through a link or QR code we provided. Participation was anonymous and voluntary, and could be paused at any point. Monetary or any other kind of compensation was not a part of our study. Contact information was also provided to parents, for sharing with us any potential concerns or worries triggered by participation.

A total of 347 Hungarian parents (of whom 82% mothers) provided complete data. Parents averaged 40.51 years old (*SD* = 7.43, range 20–75 years). Children’s mean age was 7.70 (*SD* = 2.58, range 3–12 years). The majority of participants (72.9%) reported having a college or university degree, 26.2% reported having a high-school diploma, and only 0.9% of parents marked elementary school as their highest completed education. 27.1% of parents lived in the capital, while the rest resided in other cities (47.8%) or smaller settlements (25.1%). Parents, on average, had 1.99 children (*SD* = 0.85, range 1– 6).

### 2.2 Measure

Parents filled out a demographic survey regarding their own and their child’s specifications. In case a parent had more than one child between the ages of 3 and 12, we asked them to answer all questions with the same, oldest child in mind.

Attitudes toward disability were assessed with a label-based rating task developed for the present study. Based on the definition of disabilities of the UN Convention on the Rights of Persons with Disabilities (3), we included a list of various neurodevelopmental, physical, sensory, and emotional disabilities, which contains categories describing children with special educational needs in Hungary (24). These categories overlap with those under the Individuals with Disabilities Education Act (IDEA) (29). We included the following nine disabilities: ASD, ADHD, anxiety disorders, intellectual disability, orthopedic impairment, visual impairment, hearing impairment, speech impairment, and Down syndrome. We asked parents to rate those disabilities on the five domains that comprised our study: (1) parents’ perceived knowledge of the disability (“How much do you know what it means when a child lives with [disability type]?”); (2) the perceived impact of the disability on the child’s life (“In your opinion, how much of an impact does [disability type] have on a child’s life?”); (3) parents’ attitude toward structural inclusion (“Would you agree to have a child with [disability type] go to the same group or class as your own child?”); (4) parents’ attitude toward relational inclusion (“Would you support your own child to befriend a child with [disability type]?”); and (5) the perceived ability to help an affected child or their family (“How able do you feel you are to help a child with [disability type] and their family?”). Participants answered these questions on Likert scales ranging from 1 to 3 for perceived knowledge (1= none; 2= little; 3=much), and believed impact (1= small; 2= moderate; 3= high); and from 1 to 4 for all other variables (1= not at all; 2= rather not; 3= rather yes; 4= completely yes). Moreover, we assessed whether they have had any prior experience with the above-listed disabilities, by asking the following – “yes” or “no” – question: “Have you ever had a child with [disability type] go to the same group or class as your own child?”.

### 2.3 Statistical analyses

All analyses were conducted at the item level. Descriptive statistics are reported. A series of Chi-squared tests were conducted to explore the effects of demographic variables, including parental educational level, location of residence, gender, as well as the impact of prior experiences on knowledge, perceived impact/severity, attitudes toward structural and relational inclusion and ability to help. For these comparisons, all variables were dichotomized to facilitate cross-group analyses. For *perceived knowledge* and *perceived severity/impact*, the response options “none” and “little” were combined into a single low category. For *attitudes toward structural and relational inclusion* and *perceived ability to help*, two categories were created: 0 (“not at all”/”rather not”) and 1 (“rather yes”/”completely yes”). The significance level was set at an alpha level of 0.05, using Bonferroni correction for multiple statistical tests (α’ = 0.05/k, where k is the number of statistical tests). Consequently, the level of significance was defined at 0.006 (= 0.05/9).

The effects of demographic variables, disability type and prior experience on the five attitudes were also explored in multivariate models. For each outcome, we fitted a linear mixed-effects model with the attitude-rating as the dependent variable and disability type (nine disability labels) as a within-subject fixed factor. Gender, education, place of residence, and prior experience with disability were included as between-subject fixed effects. A random intercept for participant was specified in all models to account for the non-independence of responses arising from multiple ratings given by the same individual. Although the ratings were collected on 3- and 4-point Likert-type scales, they were treated as approximately continuous in line with common practice in attitudinal research. Models were estimated using restricted maximum likelihood with a variance-components covariance structure. Statistical inference for fixed effects was based on Type III F-tests, and, where the main effect of disability type was significant, pairwise comparisons between disability categories were conducted with Sidak adjustment for multiple testing. The significance level was set at α = 0.05 for all analyses.

In a network approach, we analysed parent attitudes and perceptions toward inclusion across multiple disability types using separate symptom-agnostic networks with the same six nodes per type: Perceived Knowledge (KL), Prior Experience (PE; yes/no), Perceived Impact/Severity (IMP), Inclusion – Same Class (ICL), Inclusion – Friendship (IFR), and perceived Ability to Help (ATH). Six disability types were included in the network analysis (ASD, ADHD, anxiety disorders, intellectual disability, orthopedic impairment, and speech impairment). The other three types (visual impairment, hearing impairment, and Down syndrome) were excluded because of very low levels of prior experiences reported (~5%).

For each disability type, we estimated a regularized partial correlation network using the EBICglasso estimator in JASP (30). Because of ordinal/dichotomous items, correlations were set to Auto (polychoric for ordinal pairs; tetrachoric for dichotomous). Edges represent conditional associations between two nodes after controlling for all others. To assess stability, we ran non-parametric bootstraps (1,000 resamples) for edge accuracy and case-dropping procedures for centrality stability. We interpret strength and expected influence as centrality indices (31).

## 3 Results

### 3.1 Descriptive results

**Figure 1:**
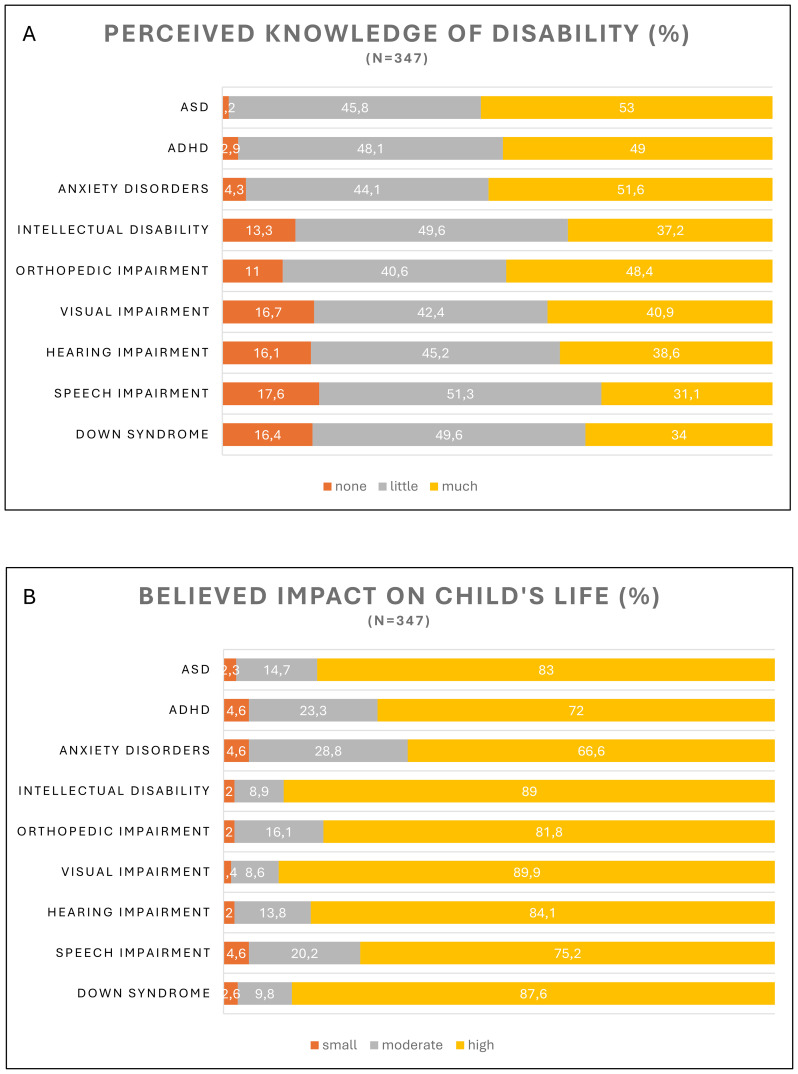
**(A)** Perceived knowledge and **(B)** impact by disability type. N = 347. ASD, autism spectrum disorder. ADHD, attention-deficit/hyperactivity disorder.

Parents reported sufficient knowledge ranging from 31.1% to 53.0% across all disability categories, with speech impairment being the least and ASD being the most well-known. About half of them reported having only a little knowledge in all categories (**Figure 1A**).

Ranging from 66.6% to 89.9%, parents reported high perceived impact across all disability categories, with anxiety disorder and visual impairment being the lowest and highest, respectively (**Figure 1B**).

**Figure 2:**
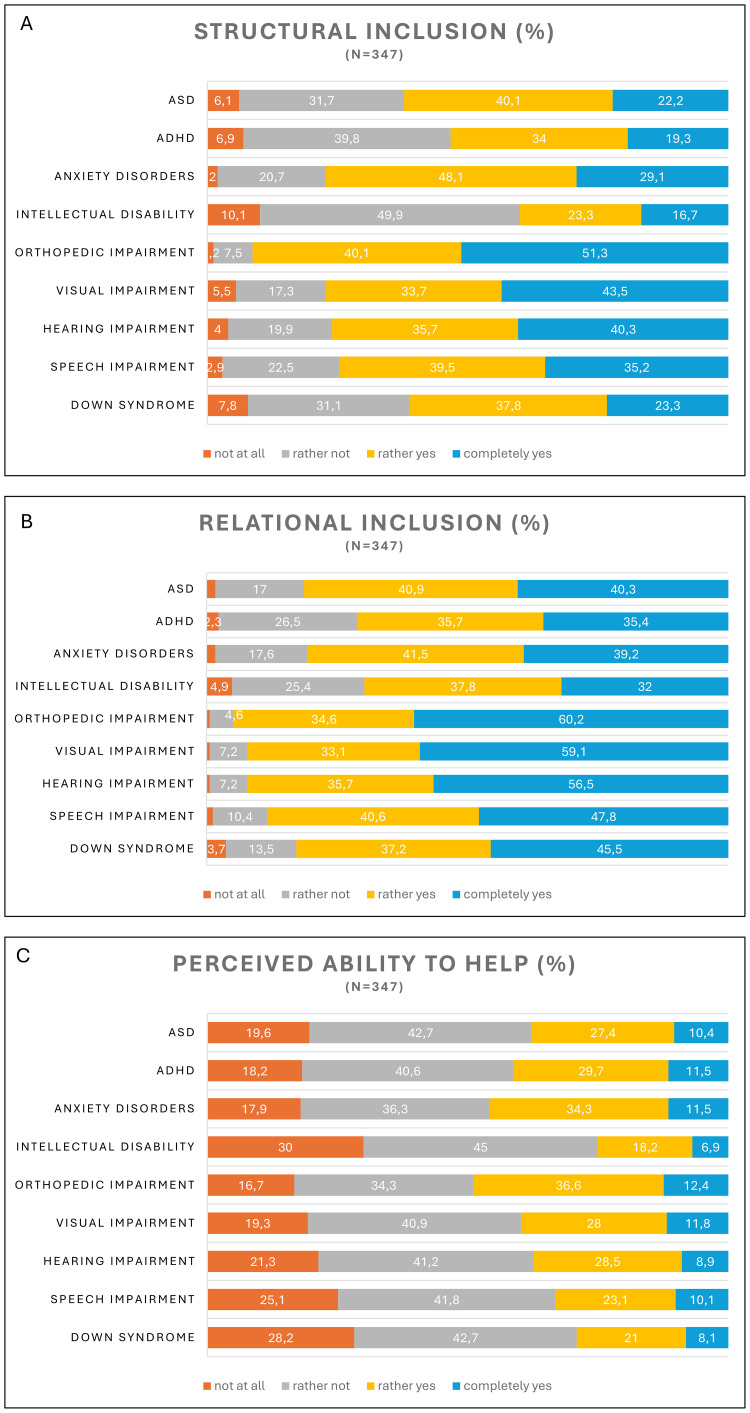
Willingness to **(A)** structural and **(B)** relational inclusion, as well as **(C)** perceived ability to help by disability type. N = 347. ASD, autism spectrum disorder. ADHD, attention-deficit/hyperactivity disorder.

Parents were the least willing to include children in the same class with intellectual disability, where overall only 40% of parents reported a positive approach. ADHD, Down syndrome, and ASD were among the least supported disabilities, with reported positive attitudes ranging from 53.3% to 62.3% (**Figure 2A**). Parents agreed to include children with orthopedic impairment in the class the most, with a cumulative 91.4%, combining “rather yes” and “completely yes” answers. Anxiety disorders were also met with a positive attitude (77.2%), while visual impairment (77.2%), hearing impairment (76%), and speech impairment (74.7%) were also given positive attitudes regarding structural inclusion.

Parents were least willing to support the friendship in the presence of ADHD, with only 35.4% giving “completely yes”, and 35.7% giving “yes” answers. Intellectual disability, with only 32% giving “completely yes”, and 37.8% giving “rather yes” answers, was also among the least supported categories in our study (**Figure 2B**). Again, the most supported category was children living with orthopedic impairment, with a cumulative positive approach of 94.8%. Similarly, parents showed high willingness to support relational inclusion regarding children living with visual impairment (92.2%), hearing impairment (92.2%), and speech impairment (88.4%).

We also assessed parents’ perceived ability to help the affected children or their families. Only a low proportion of parents were confident knowing exactly how to help, varying from 6.9% to 12.4% across the nine categories, with intellectual disability and orthopedic impairment being at the two ends of the scale (**Figure 2C**).

### 3.2 The effect of demographic variables and prior experience

Higher educational background resulted in significantly more subjective knowledge of parents concerning several included disabilities, specifically ADHD [χ^2^(2)=12.45; *p* = 0.002; Cramer’s *V* = 0.189], intellectual disability [χ^2^(2)=17.883; *p* < 0.001; Cramer’s *V* = 0.227], visual impairment [χ^2^(2)=19.112; *p* < 0.001; Cramer’s *V* = 0.235], hearing impairment [χ^2^(2)=14.622; *p* = 0.001; Cramer’s *V* = 0.205], speech impairment [χ^2^(2)=13.411; *p* = 0.001; Cramer’s *V* = 0.197], and Down syndrome [χ^2^(2)=15.067; *p* = 0.001; Cramer’s *V* = 0.208]. Moreover, parents of a higher educational background were showing more positive attitudes regarding structural inclusion for anxiety disorders [χ^2^(1)=17.687; *p* < 0.001; *Phi* = 0.226], orthopedic impairment [χ^2^(1)=8.727; *p* = 0.003; *Phi* = 0.159], visual impairment [χ^2^(1)=7.674; *p* = 0.006, *Phi* = 0.148], and Down syndrome [χ^2^(1)=8.019; *p* = 0.005; *Phi* = 0.152]. Higher educational background also meant significantly higher willingness for relational inclusion for Down syndrome [χ^2^(1)=9.692; *p* = 0.002; *Phi* = 0.167].

Participants living in the capital showed a more positive approach toward structural inclusion for ADHD [χ^2^(2)=12.2; *p* = 0.002; Cramer’s *V* = 0.188], and anxiety disorders [χ^2^(2)=23.103; *p* < 0.001; Cramer’s *V* = 0.258].

Next, we investigated whether any differences between mothers and fathers can be found regarding the five domains described above (**Supplementary Table S1**). We found that regarding the impact on a child’s life, women rated ADHD significantly higher than men [χ^2^(2)=14.099; *p* < 0.001; *Phi* = 0.202]. In terms of structural inclusion, women were significantly more supportive of children living with Down syndrome [χ^2^(2)=10.625; *p* = 0.001; *Phi* = 0.175]. In addition, mothers had a significantly more positive approach toward relational inclusion for children with the same disability [χ^2^(2)=7.724; *p* = 0.005; *Phi* = 0.149]. Moreover, women also reported having significantly more knowledge than men regarding anxiety disorders in children [χ^2^(2)=13.167; *p* = 0.001; Cramer’s *V* = 0.195].

The impact of prior experiences of participants with the included disabilities was also explored (**Supplementary Table S2**). We found that in case parents had previous experience where there had been a child with intellectual disability in the same preschool group or school class as the participant’s own child, parents showed significantly more positive attitudes toward structural inclusion [χ^2^(2)=13.545; *p* < 0.001; *Phi* = 0.198]. In addition, prior experience with some of the disabilities also resulted in parents being significantly more confident in their ability to help the affected children or their families. This was found true for ASD [χ^2^(2)=16.114; *p* < 0.001; *Phi* = 0.215], ADHD [χ^2^(2)=17.961; *p* < 0.001; *Phi* = 0.228], anxiety disorders [χ^2^(2)=33.125; *p* < 0.001; *Phi* = 0.39], and hearing impairment [χ^2^(2)=8.136; *p* = 0.004; *Phi* = 0.153]. Moreover, in cases where there had been a child with some type of disability in the class of children whose parents we asked, the parents’ perceived knowledge was significantly higher regarding ASD [χ^2^(2)=12.831; *p* = 0.002; Cramer’s *V* = 0.192], ADHD [χ^2^(2)=13.518; *p* = 0.001; Cramer’s *V* = 0.197], and anxiety disorders [χ^2^(2)=40.49; *p* < 0.001; Cramer’s *V* = 0.342].

In the multivariate models, across all five outcomes, there was a strong main effect of disability type, *F*(8, ≈2770–2774) = 17.76–86.20, *p* < 0.001, indicating systematic differences in attitudes between disability categories. Gender did not show any statistically significant main effects on the outcome variables, but there were trend-level effects. Specifically, there was a tendency for gender to be associated with perceived impact (*F*(1, 342.10) = 3.40, *p* = 0.066), perceived knowledge (*F*(1, 342.04) = 3.08, *p* = 0.080), and relational inclusion (*F*(1, 341.83) = 2.84, *p* = 0.093), such that women reported slightly more positive ratings on these items than men. Higher education was significantly associated with higher perceived knowledge and more positive attitudes toward inclusion in the same class, but not with the other attitudes. Residence was not a significant predictor in any model. In contrast, prior experience with disability was a consistent positive correlate of attitudes: participants with such experience reported higher perceived knowledge, more willingness to include peers with disabilities in the same class and as friends, and greater perceived ability to help, while perceived impact did not differ by experience (**Supplementary Tables S3**, **S4**, **Supplementary Figure S1**).

### 3.3 Results of the network analyses

**Figure 3:**
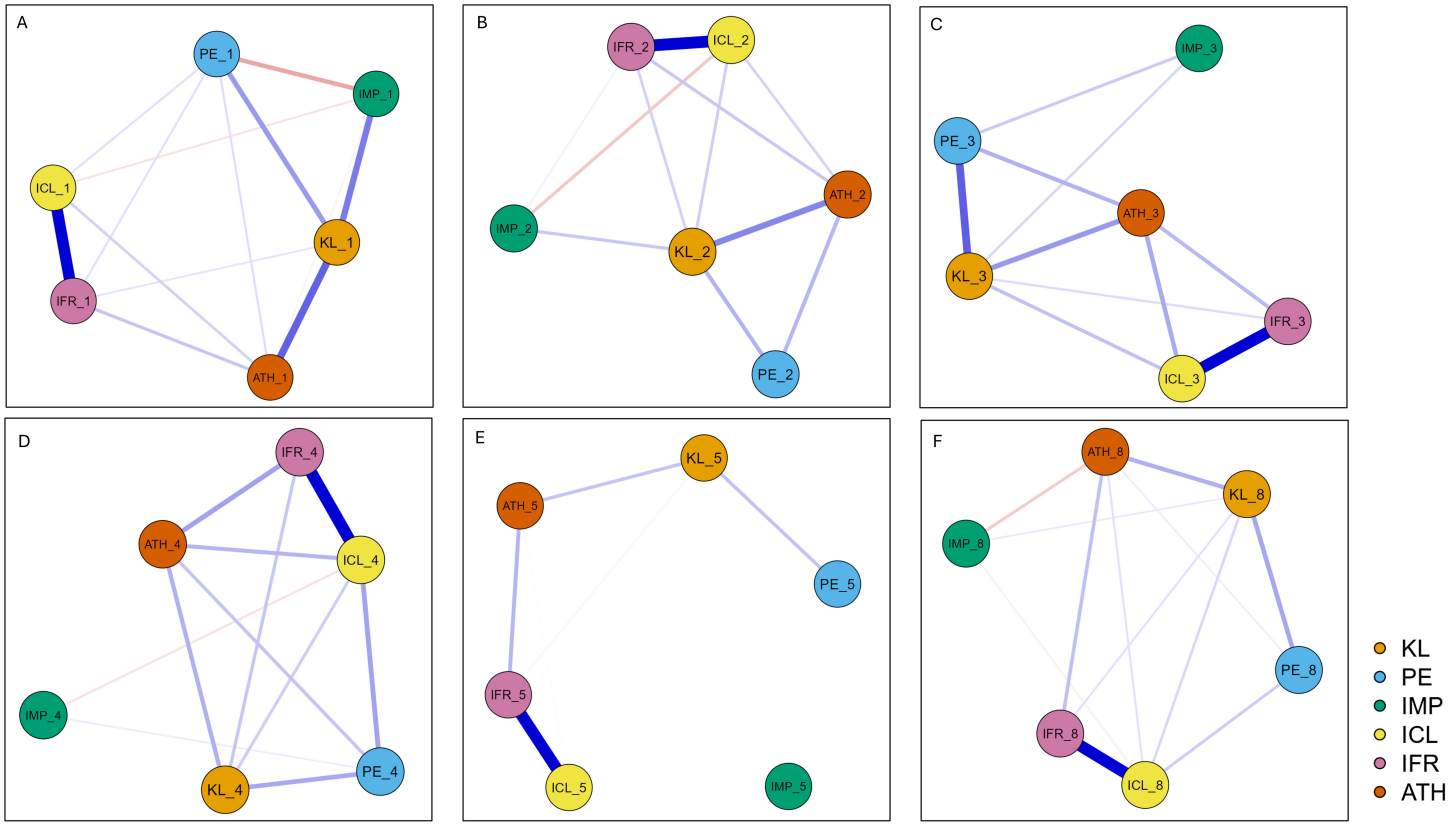
The network models. **(A)** autism spectrum disorder; **(B)** attention-deficit/hyperactivity disorder; **(C)** anxiety disorders; **(D)** intellectual disability; **(E)** orthopedic impairment; **(F)** speech impairment. *N* = 347. Blue edges represent positive relations, and red edges represent negative relations. Thicker edges indicate stronger connections. KL: perceived knowledge; IMP, perceived impact; ATH, perceived ability to help; ICL, class inclusion; IFR, friendship inclusion; PE, prior experiences.

Across all disability types, the network comprised six nodes with between 6 and 13 non-zero edges out of 15 (sparsity 0.60 to 0.13) (**Figure 3**). In every network, the ICL–IFR dyad (class inclusion ↔ friendship inclusion) emerged as one of the strongest positive edges (≈0.45–0.62), indicating a close relationship between relational and structural inclusion judgments. For most types, KL–ATH (knowledge ↔ ability to help) was moderately positive, while IMP (perceived impact) tended to have weakly negative associations with ICL (structural inclusion) or be peripheral. PE (prior experience) often showed positive edges with other nodes, especially with KL, ATH, and ICL, consistent with the idea that experience fosters knowledge and positive attitudes. Edge-wise non-parametric bootstraps indicated good accuracy; case-dropping curves declined gradually and remained positive until substantial case loss, supporting robustness of the rank order of edges. Centrality stability was acceptable (**Supplementary Material**).

The ASD network (**Figure 3A**) was the densest (13/15 non-zero edges; sparsity 0.133). The strongest edges were ICL–IFR (0.593) and KL–ATH (0.364); KL–IMP was also significantly positive (0.307). PE connected positively to KL, ICL, IFR, and ATH, and negatively to IMP (−0.199). IMP had small negative edges to ICL (−0.066) and ATH (−0.029). Based on strength centrality, KL was the most central node (1.416) in the network.

In the ADHD network (**Figure 3B**), we observed 11 of 15 non-zero edges (sparsity 0.267). Again, ICL–IFR was strong (0.570). The KL–ATH edge remained moderate (0.271), and PE–ATH was comparatively large (0.160), alongside PE–KL (0.167). IMP–ICL was negative (−0.121). ICL (1.033) had the largest strength centrality, followed by IFR (0.762).

The anxiety disorders network (**Figure 3C**) had ten of 15 non-zero edges (sparsity 0.333). The strongest positive edges connected ICL and IFR (0.527), PE and KL (0.332), as well as KL and ATH (0.210). Importantly, IMP showed no direct ties to inclusion or ability. Strength centrality was almost equally high for ICL (0.769) and KL (0.746).

In the intellectual disability network (**Figure 3D**), with 11 of 15 non-zero edges, ICL–IFR was somewhat weaker (0.449), but PE–ICL was salient (0.162) and KL–PE present (0.152). KL–ATH edge was weaker (0.139). IMP–ICL showed a mild negative edge (−0.051). Centrality clearly favoured ICL (1.323).

The orthopedic impairment network (**Figure 3E**) was the sparsest (6/15 non-zero edges; sparsity 0.600). The graph essentially hinged on ICL–IFR (0.615) with modest links IFR–ATH (0.176) and KL–ATH (0.143); KL–PE was 0.148. Most other edges were zero. Strength centrality concentrated on IFR (1.474) and ICL (0.842).

The speech impairment network (**Figure 3F**) was comparatively dense (12/15 non-zero edges; sparsity 0.200). The main positive edges were ICL–IFR (0.593), KL–ATH (0.186), and PE–ICL (0.115). Two negative edges connected IMP to ATH (−0.116) and ICL (−0.038). Again, strength centrality was highest for ICL (1.265), followed by IFR (0.890).

## 4 Discussion

While the inclusion of people with disabilities has become a defining goal in contemporary social and health policy (1), obstacles to true social participation remain widespread (6). Previous research has established that children living with disabilities face everyday challenges in social environments, especially in educational settings (7, 8). Social inclusion – or lack thereof – happens in different ecological systems that children with disabilities live in (32). Studies of social inclusion efforts underline the importance of broader contextual aspects and the role of systemic change in order to achieve a truly inclusive society (33). Verdugo and colleagues (33) believe that such a societal shift is a means for people with disabilities to be given chances for full participation, engagement and meaningful productivity, to ensure their well-being and a strong sense of belonging. In Bronfenbrenner’s framework, our study focused on mesosystems that children inhabit in their everyday lives. Although the importance of parental perspectives in shaping everyday inclusion in education has been underlined by literature (1, 2, 7, 20), there is a noticeable lack of research focusing on informal community life and everyday social interactions of children.

Moreover, despite growing attention to the inclusion of children with disabilities and their families, few studies have focused specifically on parental attitudes as a key factor. In particular, there is a lack of research comparing different types of disabilities to understand which underlying mechanisms most effectively promote positive attitudes and competent, supportive behaviors in parents. Addressing this gap could inform the development of targeted interventions, parent education programs, and broader inclusion strategies.

Therefore, the present study adopted an integrated approach to assess parents’ perceptions and attitudes towards children with nine types of disabilities across five domains: perceived knowledge, perceived impact on the child’s life, attitudes toward *structural inclusion* (participation in educational settings), attitudes toward *relational inclusion* (friendship and social interaction), and perceived ability to help affected children or families, with an additional focus on the effect of prior contact.

### 4.1 Parental perceptions

Descriptive results showed that parents in our study perceive to have moderately high knowledge on the included disabilities, with 31-53% of parents reporting to be confident in their understanding of what the given disability entails. Speech impairment, Down syndrome and intellectual disability were among the least well-known categories, while parents were the most knowledgeable about ASD. These findings are in line with previous research stating that public awareness about disabilities, such as intellectual disability, is relatively low (14). Parents reported a high perceived impact on children’s lives across all disability categories, ranging from 66.6% to 89.9%, where anxiety disorders were perceived as the least severe, and visual impairment was perceived as the most severe disability. Internalizing conditions, such as anxiety disorders, are known to be “quieter” in nature, compared to externalizing problems, often resulting in under-diagnosis (34) and a higher unawareness by surrounding adults, involving parents and teachers (35). It is important to keep in mind, however, that the health burden of such mental disorders on young people’s lives is significant: anxiety disorders were the most prevalent condition in 31 European countries among children and young adults, causing the greatest number of Years Lived with Disability – healthy life lost due to disability (36).

When investigating parental attitudes towards structural inclusion, we found larger variability compared to previous questions. Although overall attitudes were mostly accepting, intellectual disability, followed by ADHD, Down syndrome and ASD, were the least desired presence in an inclusive school setting. Similar findings were reported in previous studies, where ASD, intellectual disability, and disorders that manifest in behavioural problems were met with the least supportive attitudes regarding social inclusion (1, 14, 23, 28).

On the other hand, parents reported a salient willingness to include children in the classroom with orthopedic impairment, alongside anxiety disorders, and sensory disabilities. This is in line with previous research, suggesting that people are typically the most accepting towards individuals living with physical and sensory disabilities (1, 14, 23, 28), but somewhat contrary to findings stating that emotional problems are often met with negative attitudes in terms of social inclusion (23). However, it is important to note that emotional problems cover a broad range of conditions, from mild to severe anxiety, inhibited or avoidant behaviour, social withdrawal, emotion regulation difficulties and tantrums. We believe this contradiction in our results can be a direct consequence of different public conceptions of emotional problems (such as tantrums or conduct problems), and specific anxiety disorders, with regard to their potential social aspects, like disrupting classroom activities. In the future, terminology should be more differentiated to ensure that participants have a clear understanding of the disability in question and to gain more specific results in research.

We investigated relational inclusion as a separate aspect in our study, referring to parents’ willingness to support their own child’s friendship with another peer living with one of the disabilities we included. Results suggested very similar patterns compared to our findings regarding structural inclusion. Parents were the most supportive of orthopedic impairment and different sensory disabilities, and were the least willing to support the friendship where the child was affected by ADHD or intellectual disability, followed by a relatively low acceptance towards ASD and anxiety disorders. Bernát and colleagues (28) argue that ASD and intellectual disability are generally less accepted in society, which might have to do with how these disorders manifest in everyday life. They discuss that because both of these conditions primarily affect cognitive functioning, it might be more difficult for lay people to detect the presence of a disability, and this low awareness and familiarity lead to more negative attitudes, which also fits in the framework of intergroup contact theory (22).

We assessed the perceived ability to help children or families affected by disability, and found that across all disability categories, parents were unsure how to engage, with confident answers ranging from 6.9% to 12.4%. Participants perceived the highest self-efficacy in terms of orthopedic impairment, and were the most uncertain regarding intellectual disability. These rates are surprisingly low, especially compared to other reported measures, where parental perceptions, including knowledge and inclusion support, were high in most cases. This is a key finding of our study, as we interpret parents’ perceived ability to help as an important insufficiency in the system of inclusion implementation, potentially creating a barrier to real-life engagement.

### 4.2 The impact of prior experience

Based on the body of inclusion-related literature, we realised prior experience or contact with individuals affected by disabilities is a key factor in determining attitudes towards social inclusion. In our study, we defined prior experience as a measure of having had a child with any type of disability attended the same preschool group or school class as the participant’s own child. We found that parents with inclusion-related experience reported more positive attitudes towards the structural inclusion of children with intellectual disability. Moreover, prior experience predicted significantly more confidence in parents’ perceived ability to help in case of ASD, ADHD, anxiety disorders, and hearing impairment. Additionally, prior contact was significantly connected to higher perceived knowledge in terms of ASD, ADHD, and anxiety disorders. The effect of prior experience on attitudes remained highly significant in the multivariate analyses, after controlling for demographic variables and disability type. Similarly, previous studies established the importance of awareness and contact between disabled children and typically developing peers and families, in order for parents to form more positive attitudes towards inclusion in educational settings (1, 2, 18).

### 4.3 The effect of demographic characteristics

Our study revealed evidence suggesting higher educational background is predictive of higher subjective knowledge regarding several disabilities (ADHD, intellectual disability, visual impairment, hearing impairment, speech impairment, and Down syndrome). Moreover, a higher educational background is positively related to attitudes towards structural inclusion (anxiety disorders, orthopedic impairment, visual impairment, Down syndrome) and relational inclusion (Down syndrome). This is consistent with previous research suggesting that higher educational background is connected to more positive attitudes towards social inclusion for various disabilities (1, 2, 28), and somewhat contradicts findings that lower educational levels predicted more positive attitudes for the inclusion of children with learning disabilities (1).

More importantly, our study revealed no significant connections between the level of education and perceived self-efficacy in terms of helping children or families affected by disability, suggesting that formal education alone does not predict the practical knowledge needed to engage in real-life situations.

In terms of residence, we found that compared to participants living in other bigger cities and smaller settlements, parents living in the capital were more supportive of the structural inclusion of children with ADHD and anxiety disorders, while in other cases, we found no significant connections. This contradicts what Bernát and colleagues (28) found, according to which participants living outside the Hungarian capital reported significantly more accepting attitudes towards disabled children and adults. We interpret this contradiction as a matter of differences in sample characteristics. While Bernát and colleagues investigated the general public’s attitudes, we specifically focused on parental perceptions. It is plausible to assume that the higher rate of parents’ direct experience with disabled children not only improves inclusion-related attitudes but also weighs significantly more than demographic variables, such as place of residence alone. This claim is supported by the fact that, in multivariate models, the effect of location of residence did not reach significance.

Next, we investigated whether any gender differences appear regarding the five domains described in the study. We found that mothers reported significantly higher perceived impact/severity for ADHD, and had higher subjective knowledge of children living with anxiety disorders. In terms of structural inclusion, women were more supportive of children living with Down syndrome, and they had a more positive approach toward relational inclusion for children with the same disability. However, in multivariate analyses, the effects of gender were only apparent at the trend level. Previous research revealed rather inconsistent results; in some cases, gender played no significant role in determining inclusion-related attitudes (28), while other studies found that mothers have more positive attitudes towards some, but not all types of disabilities in children (1).

Taken together, these results suggest that demographic characteristics may have some effect, but the type of disability and the parents’ prior experiences are the key factors in attitude formation.

### 4.4 Network analyses

To explore the specific structure of parental perceptions and attitudes towards different disability types, we conducted six separate network analyses.

The ASD network was the densest and most coherent graph. The strong structural and relational inclusion dyad indicated that the two types of inclusion judgments move almost in lockstep. Equally striking was the connection between perceived knowledge and ability to help, suggesting that parents who feel they know what the disability entails also feel *able to help*. The structure of the network also indicated that people who read/learn more also notice more impact; however, perceived impact’s small negatives to structural inclusion attitudes and perceived ability to help suggest that unbuffered severity perception can dampen inclusion and efficacy. Prior experience was proven to be broadly helpful and modestly linked to inclusion and ability. Centrality indices suggested that this network is knowledge-led. Thus, in line with previous findings (13), targeted psychoeducation that provides parents and children with specific, practical knowledge (brief, concrete information about autistic characteristics and everyday support strategies) is key in order to achieve reduced negative attitudes and improve the willingness and ability to engage in everyday situations where help is needed. In the ADHD network, the structural and relational inclusion dyad was again strong, but the standout feature is that prior experience was unusually predictive of feeling capable, even more than knowledge per se. Prior experience was also connected to perceived knowledge, creating a contact→knowledge→ability chain. At the same time, the negative edge between perceived impact and structural inclusion indicated that, when impact/severity is perceived to be high, support for same-class placement weakens. In this network, social-norm/relationship considerations were the organizing themes, with experience acting as the most efficient lever on capability. This finding highlights the importance of guided peer contact in and outside of school settings, in order for children with ADHD to have more opportunities for meaningful social participation. Structured, positive contact with typically developing children, rather than mere co-presence, helps form more positive attitudes and a willingness to initiate social interactions (21). It is suggested that system-level norms and interventions, such as cooperative learning, peer tutoring, or buddy systems, should be introduced in inclusive school settings to provide guided contact between students with ADHD and their peers (37). These activities that make ADHD-related strengths visible may help reframe “disruptive” behaviour and support more stable, positive attitudes. The edge between prior experience and perceived knowledge was found to be the strongest in the anxiety disorders network, and the connection between knowledge and ability to help was also clearly positive. The core of the network remained the dyad of structural and relational inclusion, but influence radiated from perceived knowledge and attitudes toward structural inclusion in parallel, indicating that building experience that grows knowledge looks like the key route. On the other hand, reframing “impact” is less critical here. As mentioned earlier, Rademaker and colleagues (10) demonstrated that providing personal contact and disability-specific knowledge together brought the most convincing results in the attitude formation of peers, improving the quality of social participation of students with disabilities. Moreover, because of the quieter nature of internalizing problems, it is typically harder for others with different thresholds for problem detection to recognize them (38). Therefore, in this case, preventive education involving parents and children could be key in improving awareness and the ability to help. Interventions that increase the visibility of internalizing problems (e.g., psychoeducation on “invisible worries”) and teach simple, supportive responses could be especially effective in strengthening both helping intentions and inclusive attitudes. In the intellectual disability network, the structural and relational inclusion tie is a touch weaker than usual; however, real-world contact appears to translate directly into comfort with same-class inclusion. Knowledge still helps ability, but higher impact judgments are associated with lower levels of willingness to structural inclusion. According to centrality indices, structural inclusion attitudes organize the network; the best entry point is contact that normalizes the classroom, plus light severity reframing. Although children with special educational needs in Hungary have the possibility to attend institutions partly or fully integrated with other peers (27), current policy typically places students with intellectual disability in segregated or partly segregated institutions, appointed by the professional diagnostic committee. This policy and high rate of segregation may result in parents’ negative attitude towards integration in mainstream schools, resulting in the centrality of structural inclusion in the network. On the other hand, forty percent of parents in our study reported positive attitudes regarding educational inclusion, perhaps reflecting more openness than systemic policies allow. The pattern points to the need for interventions that simultaneously reframe impact (e.g., emphasizing what is possible with support rather than global “severity”) and create repeated, guided contact experiences in which children with intellectual disability can demonstrate meaningful participation in everyday classroom and community roles.

The orthopedic impairment network is the sparsest structure: beyond the strongest structural-relational inclusion connection, only the connections between prior experiences and perceived knowledge, perceived knowledge and ability to help, as well as relational inclusion and ability to help were significant. All other partial edges were shrunk to zero. Centrality concentrated on friendship inclusion willingness, implying that friendship and everyday social contact are prerequisites; without them, knowledge and experience do not integrate into a broader pro-inclusion pattern. Here, the priority is to seed relationships (buddying, structured play) and then grow capability. Research has established that among the different disability types, children living with orthopedic impairment are generally the least stigmatized group and typically meet openness for inclusion; however, it is very important to keep in mind the significance of personal experience and awareness. To form positive attitudes towards structural and relational inclusion, and to grow parents’ and peers’ confidence in their ability to help children with orthopedic impairment, ensuring possibilities to build contact and peer relationships is of high importance (2, 21).

The speech impairment network was shown to be a dense, typical configuration: a very strong connection between structural and friendship inclusion willingness, positive edges between perceived knowledge and ability to help, as well as prior experience and structural inclusion, indicating that contact appears to normalize mainstream placement directly. Two negative edges of impact ratings to the ability to help and structural inclusion are noteworthy; here, perceived impact clearly pushes against both helping and placement. In that case, capability building and severity reframing could be equally important. Targeted interventions that normalise speech differences, teach simple communication strategies to children and parents, and clearly separate speech problems from cognitive ability may reduce perceived burden and strengthen both inclusion and helping intentions. There is some evidence in the literature that connects teachers’ perceived severity to inclusion willingness; a review found that in the case of mild disabilities, they showed significantly more positive attitudes towards classroom inclusion, as opposed to more complex needs and externalizing symptoms (39). Although the present study exclusively investigated parental attitudes, teachers are equally significant agents in forming inclusive school settings that enable children with disabilities to experience full social participation (1, 7).

### 4.5 Clinical implications

Overall, three conclusions can be generalized across types of disabilities. First, the results suggest that relational and structural inclusion are strongly connected, indicating that interventions that foster friendships plausibly shift class inclusion attitudes, and vice versa. This finding highlights the importance of creating opportunities for everyday interaction between typically and atypically developing children and families outside of institutional settings, as a way of ensuring relational acceptance to find its way into inclusive classrooms. Second, the association between perceived knowledge and self-efficacy to help is reliably positive, indicating that greater perceived understanding tends to translate into greater helping capability. However, the effect size is heterogeneous, suggesting that its magnitude varies by context (e.g., disability type, prior contact, educational level, and measurement). Third, while experience usually helps, perceived impact/severity ranges from benign/peripheral (anxiety disorders, orthopedic impairment) to mildly antagonistic toward inclusion and helping (ADHD, intellectual disability, speech impairment).

Though the network analyses revealed that a specific actionable pathway applies to all disability types (contact → knowledge/capability → inclusion), the entry point varies, with a focus on knowledge (ASD), guided contact (ADHD), contact-to-knowledge pipelines (anxiety disorders), impact reframing plus contact (intellectual disability and speech impairment), and relationship seeding (orthopedic impairment). These findings reveal that although some elements of a successful inclusion implementation process are universal, taking the specific disability into account is of high importance, and could inform policy makers and professionals working in inclusive settings.

Moreover, a review (40) concluded that most interventions aim at intrapersonal aspects, such as teaching social skills to children with disabilities, rather than applying a systemic approach. Our results suggest that universal and disability-specific system-level interventions are just as important. There is evidence that direct and indirect contact-based interventions in school settings significantly improve attitudes towards disability (41). Examples include role-playing activities that promote problem-solving, and the use of materials such as books, films, short stories, or puppets, followed by discussions. For direct contact-based interventions, Lindner and colleagues (37) suggest cooperative learning, peer tutoring, and buddy systems. Our finding that structural and relational inclusion willingness are strongly correlated suggests that inclusion should not be limited to the school environment. Attitudes may be effectively shaped by contact opportunities outside of educational settings, e.g. accessible playgrounds (42–45) and inclusive community events for families involving opportunities for sports, art, and other leisure activities for all. Preliminary evidence from Hungary suggests that structured contact in shared play environments can positively affect intergroup relations between children with and without disabilities, underscoring the potential of inclusive playgrounds and similar community spaces as attitude-shaping contexts (42).

For enhancing positive inclusion attitudes through improving knowledge, we suggest targeted psychoeducation for parents and teachers, providing disability-specific practical knowledge, as a means of raising awareness and reframing perceived impact, by informing participants about treatment options and ways they can help the affected individuals. Tools like Mental Health First Aid (46) are proven to better public mental health literacy and improve self-efficacy in helping those in need (13). In the Hungarian context, school-based sensitisation and awareness-raising programmes have also been shown to improve disability-related attitudes among students and teachers, supporting the promise of combined contact- and knowledge-based approaches (47, 48).

According to UNICEF (49) it is important to note that, when creating and implementing inclusive interventions, it is beneficial to work together with disabled children and their families, as their experiences, opinions and needs should be reflected in inclusion efforts. They add that domains like family and community life (e.g. sports, culture, recreation) lack evidence in inclusion research (49).

### 4.6 Limitations

We realise that the present study is not without limitations. First, our sample is not representative of the general population, given that more than eighty per cent of our participants were mothers, and the majority of parents had a college or university degree. Future work should put more focus on the role of fathers in developing and implementing inclusion-related practices in and outside of educational settings, as well as investigating the perceptions and attitudes of parents with an average or lower educational background, to gain a more generalisable picture of the state of disabled children’s social participation.

Despite the acknowledgement of Bernát and colleagues’ (28) noteworthy finding that participant responses in attitude research may be influenced by social desirability, our study challenges this assertion. We contend that the substantial variability in perceptions and attitudes across diverse disability types observed in our research renders this concern moot. In addition, it is challenging to ascertain the extent to which the attitudes measured in this study would translate into real-life behaviors. Another potentially important aspect is whether the participating parents’ children, or perhaps the parents themselves, are affected by some type of disability. In the present study, the personal impact was not taken into statistical consideration, which precludes the ability to draw conclusions based on that factor; however, in future studies, it would be beneficial to take this important aspect of attitude formation into account.

Furthermore, all constructs were assessed through single-item measures, which restricts reliability estimation and may not fully capture the complexity of each domain. Future work should employ multi-item scales with established psychometric properties to enhance internal consistency. The online questionnaire format raises concerns regarding survey fraud, respondent fatigue, and the lack of possibility for clarification. To reach a broader population and get a deeper understanding of attitude and perception formation towards inclusion, future research should also examine qualitative information from parents, even in smaller settlements, where a lack of internet access would otherwise hinder the possibility of participation. In addition, we realize that perceived knowledge measured here is not equivalent to objective knowledge, as misconceptions and stereotypes may influence these self-ratings. Qualitative measures could make it possible to assess factual, rather than perceived parental knowledge of disabilities.

Moreover, during the conduct of the network analyses, we had to dismiss three disability categories due to very low percentages of reported prior experience. In the future, it would be very important to assess parental perceptions regarding those disabilities more thoroughly, perhaps with a more targeted sample.

A further limitation of the present network analysis is its correlational nature, which precludes causal inference. Some relationships may be bidirectional; for example, individuals who are more active in helping may subsequently acquire greater knowledge. Moreover, shared method variance may inflate associations. Longitudinal or intervention-based designs, integrating contact-based and psychoeducational components, would allow stronger causal conclusions and inform evidence-based parental engagement strategies. Future studies should also include moderator analyses (e.g., educational level) to better understand the contextual factors shaping these relationships.

### 4.7 Conclusion

In a community sample of Hungarian parents, attitudes toward both structural (same-class) and relational (friendship) inclusion were generally positive across disability types. Yet, self-efficacy to help remained strikingly low, a potential barrier to inclusion. Network analyses consistently linked friendship and class inclusion, and — albeit with heterogeneous effect sizes — perceived knowledge to helping capability, while perceived impact/severity showed diagnosis-specific dampening of inclusion and helping (notably for ADHD, intellectual disability and speech impairment).

Together with external evidence from intergroup contact theory, these patterns suggest an actionable pathway (contact → knowledge/capability → inclusion), with the optimal entry point varying by condition (e.g., knowledge-led for ASD; guided contact for ADHD; severity-reframing plus contact for intellectual disability and speech impairment). Future work should deploy multicomponent interventions that combine positive contact and disability-specific psychoeducation, test causal mechanisms in longitudinal/experimental designs and use reliable multi-item measures with harmonized scales.

## Data Availability

The datasets presented in this study can be found in online repositories. The names of the repository/repositories and accession number(s) can be found below: The datasets analyzed for this study have been uploaded and are freely available in the OSF: https://osf.io/k3ecm/overview?view_only=be582875e0134806a31f3113da12384f.
