# Supplementary material for: Parental perceptions and attitudes towards the inclusion of children with neurodevelopmental, physical and sensory disabilities
Source: Front Psychiatry. 2026 Jan 20;16:1735746. doi: 10.3389/fpsyt.2025.1735746 (PMC12864138; doi:10.3389/fpsyt.2025.1735746)

Supplementary Material

Kárpáti, N. & Miklósi, M. (2026) Parental perceptions and attitudes towards the inclusion of children with neurodevelopmental, physical and sensory disabilities. Front. Psychiatry16:1735746. doi:10.3389/fpsyt.2025.1735746

Table S1. Gender differences

Gender differences	(Rather high) effect on a child's life					Structural inclusion					Relational inclusion					Ability to help				
	Women (%)	Men (%)	χ^2	p	Phi	Women (%)	Men (%)	χ^2	p	Phi	Women (%)	Men (%)	χ^2	p	Phi	Women (%)	Men (%)	χ^2	p	Phi
ASD	**85**	**73.8**	**4.465**	**0.035**	**0.113**	63.6	55.7	1.335	0.248	0.062	**83.6**	**70.5**	**5.646**	**0.017**	**0.128**	**40.9**	**23**	**6.9**	**0.009**	**0.141**
ADHD	**76.2**	**52.5**	**14.099**	**<0.001**	**0.202**	53.1	54.1	0.018	0.892	-0.007	72.4	65.6	1.135	0.287	0.057	**43.7**	**29.5**	**4.183**	**0.041**	**0.11**
Anxiety disorder	68.5	57.4	2.811	0.094	0.09	78.3	72.1	1.096	0.295	0.056	**83.2**	**68.9**	**6.658**	**0.01**	**0.139**	**49**	**31.1**	**6.419**	**0.011**	**0.136**
Intellectual disability	**90.9**	**80.3**	**5.772**	**0.016**	**0.129**	40.9	36.1	0.491	0.483	0.038	**72.4**	**57.4**	**5.361**	**0.021**	**0.124**	**28**	**11.5**	**7.283**	**0.007**	**0.145**
Orthopedic impairment	80.8	86.9	1.266	0.261	-0.06	92.7	85.2	3.497	0.061	0.1	**96.2**	**88.5**	**5.95**	**0.015**	**0.131**	49.3	47.5	0.062	0.803	0.013
Visual impairment	89.5	91.8	0.291	0.589	-0.029	78	73.8	0.505	0.477	0.038	93.4	86.9	2.934	0.087	0.092	39.9	39.3	0.006	0.94	0.004
Hearing impairment	84.6	82	0.264	0.607	0.028	77.6	68.9	2.125	0.145	0.078	93.4	86.9	2.934	0.087	0.092	37.8	36.1	0.062	0.804	0.013
Speech impairment	**77.3**	**65.6**	**3.691**	**0.055**	**0.103**	75.9	68.9	1.31	0.252	0.061	89.2	85.2	0.756	0.385	0.047	34.6	26.2	1.596	0.207	0.068
Down-syndrome	88.8	82	2.169	0.141	0.079	**65**	**42.6**	**10.625**	**0.001**	**0.175**	**85.3**	**70.5**	**7.724**	**0.005**	**0.149**	**31.5**	**18**	**4.398**	**0.036**	**0.113**

Notes. N= 347. ASD: autism spectrum disorder. ADHD: attention-deficit/hyperactivity disorder.

Table S2. The effect of Prior experience

Prior experience	(Rather high) effect on child's life					Structural inclusion					Relational inclusion					Ability to help				
	Yes (%)	No (%)	χ2	p	Phi	Yes (%)	No (%)	χ2	p	Phi	Yes (%)	No (%)	χ2	p	Phi	Yes (%)	No (%)	χ2	p	Phi
ASD	78.6	85.5	2.746	0.098	-0.089	**69**	**58.4**	**3.893**	**0.049**	**0.106**	85.7	78.7	2.569	0.109	0.086	**51.6**	**29.9**	**16.114**	**<0.001**	**0.215**
ADHD	72.6	71.1	0.91	0.762	0.016	54.3	51.6	0.25	0.617	0.027	74.4	65.6	3.053	0.081	0.094	**49.8**	**26.6**	**17.961**	**<0.001**	**0.228**
Anxiety disorder	**73.4**	**60.8**	**6.111**	**0.013**	**0.133**	**85.4**	**70.4**	**11.119**	**0.001**	**0.179**	84.8	77.2	3.158	0.076	0.095	**62.7**	**31.7**	**33.125**	**<0.001**	**0.39**
Intellectual disability	90.3	88.9	0.057	0.812	0.013	**71**	**37**	**13.545**	**<0.001**	**0.198**	77.4	69	0.951	0.329	0.052	**45.2**	**23.1**	**7.313**	**0.007**	**0.145**
Orthopedic impairment	74.3	82.7	1.497	0.221	-0.066	94.3	91	0.423	0.515	0.035	91.4	95.2	0.906	0.341	-0.051	60	47.8	1.888	0.169	0.074
Visual impairment	85.7	90	0.139	0.709	-0.02	85.7	77.1	0.292	0.589	0.029	85.7	92.4	0.421	0.516	-0.035	57.1	39.4	0.9	0.343	0.051
Hearing impairment	85.7	84	0.041	0.84	0.011	**95.2**	**74.8**	**4.508**	**0.034**	**0.114**	100	91.7	1.886	0.17	0.074	**66.7**	**35.6**	**8.136**	**0.004**	**0.153**
Speech impairment	69.8	76	0.782	0.377	-0.047	83.7	73.4	2.138	0.144	0.079	**97.7**	**87.2**	**4.075**	**0.044**	**0.108**	41.9	31.9	1.684	0.194	0.07
Down-syndrome	100	87	2.373	0.123	0.083	81.3	60.1	2.867	0.09	0.091	87.5	82.5	0.269	0.604	0.028	37.5	28.7	0.573	0.449	0.041

Notes. N= 347. ASD: autism spectrum disorder. ADHD: attention-deficit/hyperactivity disorder.

Table S3. Results of the Linear Mixed-Effects Models (Type III Tests of Fixed Effects)

Attitude type (dependent variable)		df1	df2	*F*	*p*
Perceived Knowledge	Intercept	1	372.189	3798.396	<0.001
	Disability Type	8	2772.363	17.756	<0.001
	Gender	1	342.039	3.084	0.080
	Education	1	342.151	18.302	<0.001
	Location of Residence	2	342.003	0.755	0.471
	Prior Experience	1	2901.633	93.834	<0.001
Perceived Impact	Intercept	1	383.136	11293.651	<0.001
	Disability Type	8	2774.244	20.141	<0.001
	Gender	1	342.099	3.404	0.066
	Education	1	342.251	3.379	0.067
	Location of Residence	2	342.050	0.860	0.424
	Prior Experience	1	2944.482	0.191	0.662
Structural Incusion	Intercept	1	374.742	3619.770	<0.001
	Disability Type	8	2772.577	86.202	<0.001
	Gender	1	341.778	2.509	0.114
	Education	1	341.900	6.570	0.011
	Location of Residence	2	341.739	2.447	0.088
	Prior Experience	1	2912.964	10.273	0.001
Relational Inclusion	Intercept	1	363.550	4758.294	<0.001
	Disability Type	8	2770.696	63.562	<0.001
	Gender	1	341.826	2.840	0.093
	Education	1	341.907	1.919	0.167
	Location of Residence	2	341.800	1.216	0.298
	Prior Experience	1	2866.080	4.796	0.029
Perceived Ability to Help	Intercept	1	360.415	1562.198	<0.001
	Disability Type	8	2770.065	19.726	<0.001
	Gender	1	341.723	2.360	0.125
	Education	1	341.793	0.005	0.945
	Location of Residence	2	341.700	0.530	0.589
	Prior Experience	1	2852.809	52.992	<0.001

Notes. N= 347.

Figure S1. Attitudes Across Disability Type

Notes. N=347. Estimated Marginal Means, Controlling for Gender, Education, Location of Residence, and Prior Experience. ASD: autism spectrum disorder. ADHD: attention-deficit/hyperactivity disorder.

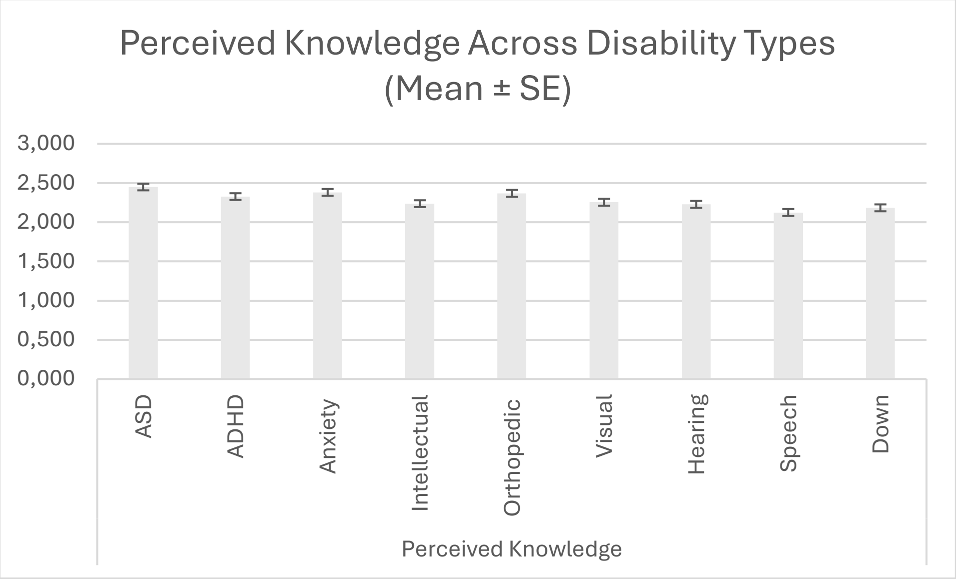

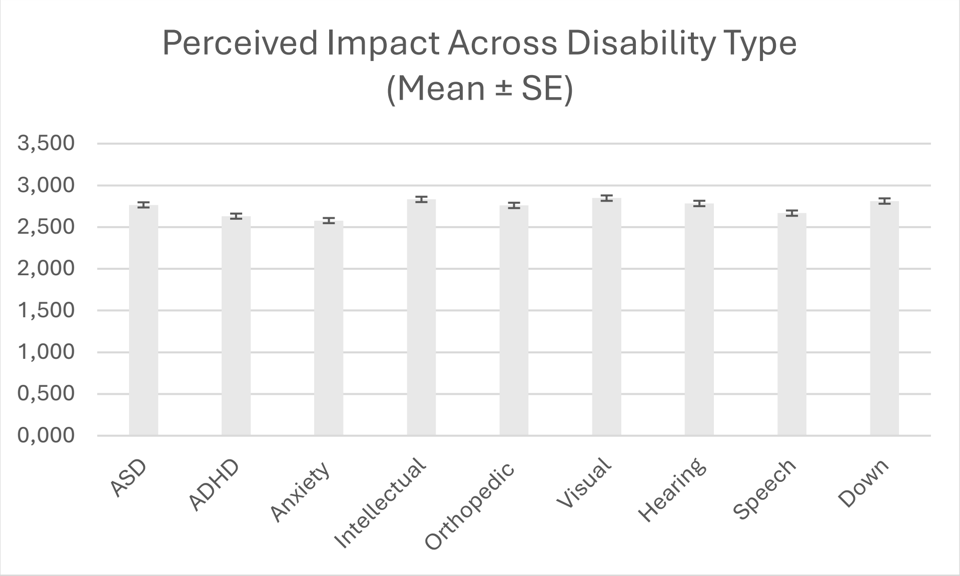

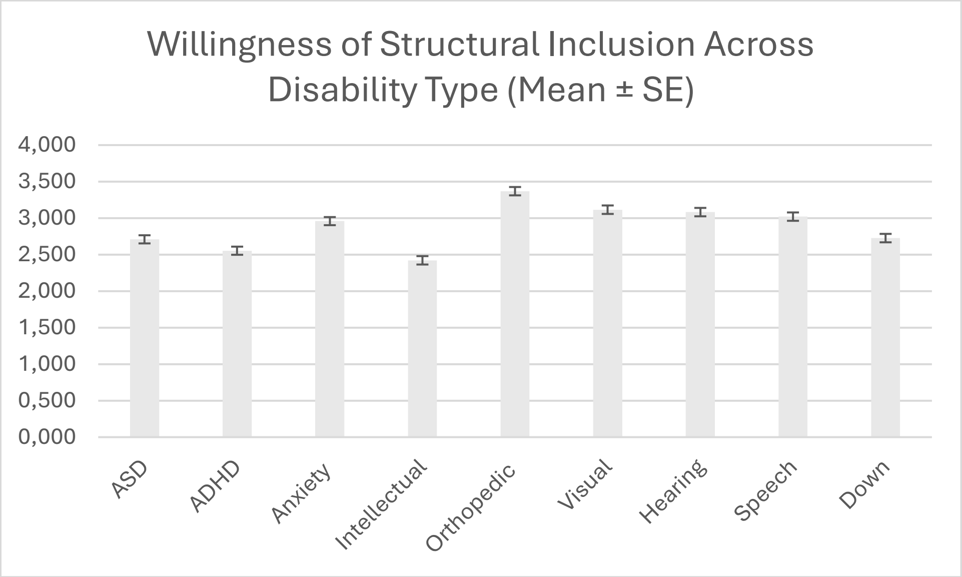
Willingness of Structural Inclusion Across Disability Type (Mean ± SE)
4,000
3,500
3,000
2,500
2,000
1,500
1,000
0,500
0,000
ASD
ADHD
Anxiety
Intellectual
Orthopedic
Visual
Hearing
Speech
Down

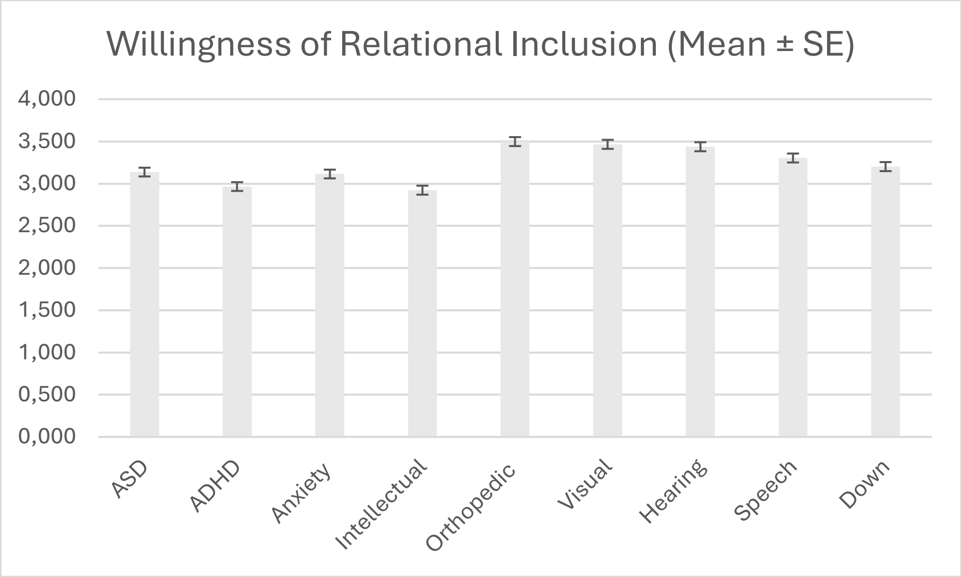
Willingness of Relational Inclusion (Mean ± SE)
4,000
3,500
3,000
2,500
2,000
1,500
1,000
0,500
0,000
ASD
ADHD
Anxiety
Intellectual
Orthopedic
Visual
Hearing
Speech
Down

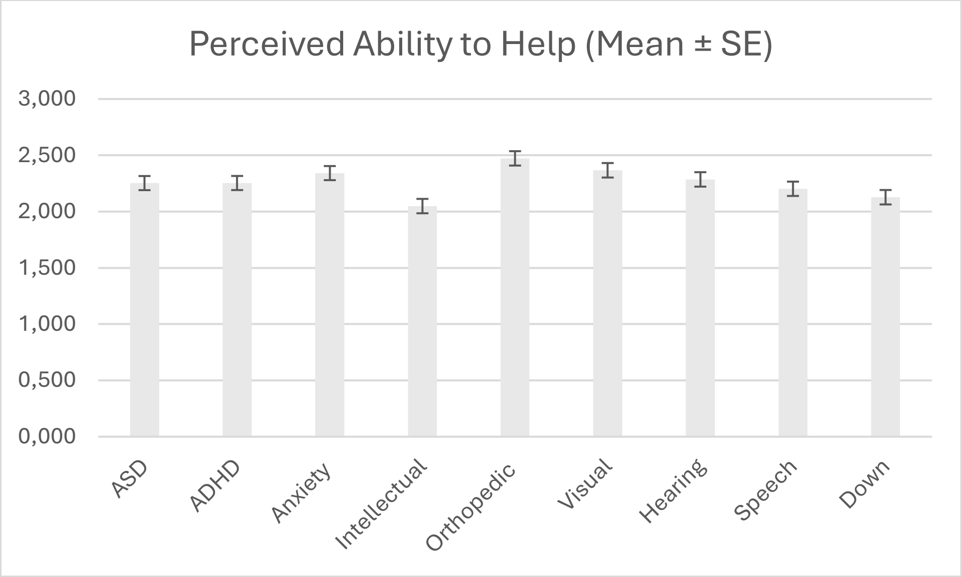
Perceived Ability to Help (Mean ± SE)
3,000
2,500
2,000
1,500
1,000
0,500
0,000
ASD
ADHD
Anxiety
Intellectual
Orthopedic
Visual
Hearing
Speech
Down

Table S4. Estimated Marginal Means

Notes. N=347. Estimated Marginal Means, Controlling for Gender, Education, Location of Residence, and Prior Experience. ASD: autism spectrum disorder. ADHD: attention-deficit/hyperactivity disorder.

Attitude Type	Disability Type	Mean	SE	df	95% CI	
Perceived Knowledge	ASD	2,450	0,043	643,532	2,366	2,533
	ADHD	2,327	0,043	639,453	2,243	2,411
	Anxiety	2,381	0,043	635,677	2,298	2,465
	Intellectual	2,236	0,044	707,600	2,150	2,323
	Orthopedic	2,369	0,044	703,642	2,283	2,455
	Visual	2,256	0,044	733,744	2,169	2,343
	Hearing	2,229	0,044	717,993	2,143	2,315
	Speech	2,124	0,044	696,064	2,039	2,210
	Down	2,184	0,044	723,456	2,097	2,270
Perceived Impact	ASD	2,766	0,031	773,955	2,705	2,827
	ADHD	2,631	0,031	768,210	2,570	2,692
	Anxiety	2,578	0,031	762,890	2,517	2,638
	Intellectual	2,832	0,032	863,797	2,769	2,895
	Orthopedic	2,760	0,032	858,269	2,697	2,822
	Visual	2,847	0,032	900,220	2,783	2,910
	Hearing	2,783	0,032	878,294	2,720	2,846
	Speech	2,667	0,032	847,678	2,605	2,730
	Down	2,812	0,032	885,906	2,749	2,875
Structural Inclusion	ASD	2,709	0,056	676,044	2,600	2,819
	ADHD	2,553	0,056	671,534	2,444	2,663
	Anxiety	2,959	0,056	667,359	2,849	3,068
	Intellectual	2,422	0,057	746,827	2,309	2,535
	Orthopedic	3,369	0,057	742,457	3,256	3,481
	Visual	3,115	0,058	775,677	3,001	3,229
	Hearing	3,082	0,058	758,298	2,969	3,195
	Speech	3,020	0,057	734,090	2,908	3,133
	Down	2,726	0,058	764,328	2,613	2,840
Relational Inclusion	ASD	3,137	0,052	549,843	3,035	3,239
	ADHD	2,966	0,052	547,022	2,864	3,068
	Anxiety	3,114	0,052	544,411	3,013	3,216
	Intellectual	2,923	0,053	594,165	2,819	3,027
	Orthopedic	3,498	0,053	591,425	3,394	3,603
	Visual	3,466	0,054	612,274	3,361	3,571
	Hearing	3,437	0,053	601,362	3,333	3,542
	Speech	3,304	0,053	586,180	3,200	3,408
	Down	3,202	0,053	605,146	3,097	3,307
Perceived Ability to Help	ASD	2,253	0,063	517,782	2,130	2,376
	ADHD	2,254	0,063	515,398	2,131	2,377
	Anxiety	2,342	0,063	513,191	2,219	2,465
	Intellectual	2,049	0,064	555,216	1,923	2,174
	Orthopedic	2,473	0,064	552,902	2,347	2,598
	Visual	2,367	0,064	570,503	2,240	2,493
	Hearing	2,286	0,064	561,291	2,160	2,412
	Speech	2,202	0,064	548,474	2,077	2,328
	Down	2,128	0,064	564,486	2,001	2,254

Attitude Type	Gender	Mean	SE	df	95% CI	
Perceived Knowledge	Women	2,343	0,033	376,140	2,278	2,409
	Men	2,225	0,063	353,174	2,102	2,348
Perceived Impact	Women	2,785	0,023	388,543	2,739	2,830
	Men	2,699	0,043	357,206	2,613	2,784
Structural Inclusion	Women	2,953	0,043	379,068	2,868	3,038
	Men	2,815	0,081	353,941	2,656	2,974
Relational Inclusion	Women	3,300	0,042	366,384	3,217	3,382
	Men	3,155	0,079	349,871	2,999	3,311
Ability to Help	Women	2,342	0,051	362,849	2,241	2,443
	Men	2,181	0,097	348,652	1,990	2,372

Attitude Type	Education	Mean	SE	df	95% CI	
Perceived Knowlegde	low/medium	2,160	0,054	357,744	2,054	2,266
	high	2,408	0,039	365,263	2,331	2,486
Perceived Impact	low/medium	2,705	0,037	363,424	2,631	2,778
	high	2,779	0,027	373,674	2,725	2,832
Structural Inclusion	low/medium	2,788	0,070	358,938	2,651	2,925
	high	2,980	0,051	367,162	2,880	3,080
Relational Inclusion	low/medium	3,176	0,068	353,166	3,042	3,310
	high	3,279	0,050	358,577	3,181	3,376
Ability to Help	low/medium	2,265	0,083	351,487	2,101	2,428
	high	2,258	0,061	356,141	2,139	2,378

Attitude Type	Residence	Mean	SE	df	95% CI	
Perceived Knowledge	capital	2,242	0,056	353,478	2,132	2,352
	city	2,318	0,045	363,375	2,229	2,407
	smaller settlement	2,292	0,057	354,675	2,180	2,405
Perceived Impact	capital	2,715	0,039	357,618	2,639	2,791
	city	2,733	0,031	371,097	2,671	2,795
	smaller settlement	2,777	0,040	359,247	2,700	2,855
Structural Inclusion	capital	2,989	0,072	354,273	2,847	3,131
	city	2,878	0,059	365,096	2,762	2,993
	smaller settlement	2,786	0,074	355,582	2,641	2,931
Relational Inclusion	capital	3,302	0,071	350,090	3,163	3,441
	city	3,219	0,057	357,219	3,106	3,331
	smaller settlement	3,162	0,072	350,954	3,020	3,304
Ability to Help	capital	2,242	0,087	348,841	2,072	2,412
	city	2,316	0,070	354,973	2,178	2,454
	smaller settlement	2,226	0,088	349,584	2,053	2,400

Attitude Type	Prior Experience	Mean	SE	df	95% CI	
Perceived Knowledge	No	2,163	0,037	353,951	2,091	2,235
	Yes	2,405	0,041	570,474	2,324	2,487
Perceived Impact	No	2,737	0,025	358,263	2,687	2,787
	Yes	2,746	0,030	658,653	2,687	2,805
Structural Inclusion	No	2,830	0,047	354,791	2,737	2,923
	Yes	2,938	0,054	592,885	2,832	3,045
Relational Inclusion	No	3,198	0,046	350,432	3,107	3,289
	Yes	3,257	0,051	503,404	3,157	3,357
Ability to Help	No	2,150	0,057	349,135	2,038	2,262
	Yes	2,373	0,062	479,682	2,252	2,494

Network Analysis 1: Autism spectrum disorder

Summary of Network

Number of nodes	Number of non-zero edges	Sparsity
6	13 / 15	0.133

Centrality measures per variable

	Network			
**Variable**	**Betweenness**	**Closeness**	**Strength**	**Expected influence**
KL_1	1.107	1.240	1.416	1.072
PE_1	-0.886	-0.466	-0.865	-0.880
IMP_1	-0.886	-0.244	-1.190	-1.520
ICL_1	-0.886	-1.091	0.466	0.350
IFR_1	0.443	-0.678	0.634	0.775
ATH_1	1.107	1.240	-0.461	0.203

Weights matrix

	Network					
**Variable**	**KL_1**	**PE_1**	**IMP_1**	**ICL_1**	**IFR_1**	**ATH_1**
KL_1	0.000	0.234	0.307	0.000	0.060	0.364
PE_1	0.234	0.000	-0.199	0.069	0.070	0.073
IMP_1	0.307	-0.199	0.000	-0.066	0.000	-0.029
ICL_1	0.000	0.069	-0.066	0.000	0.593	0.104
IFR_1	0.060	0.070	0.000	0.593	0.000	0.133
ATH_1	0.364	0.073	-0.029	0.104	0.133	0.000

Network

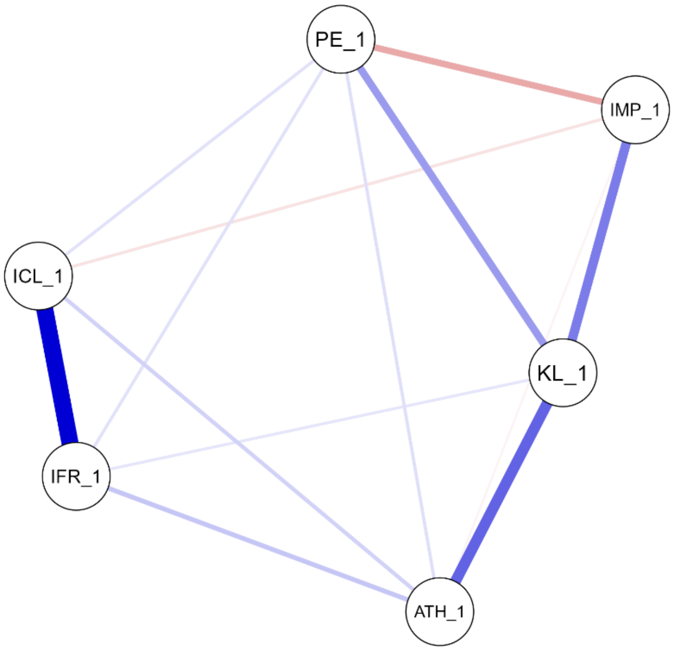

Centrality Plot

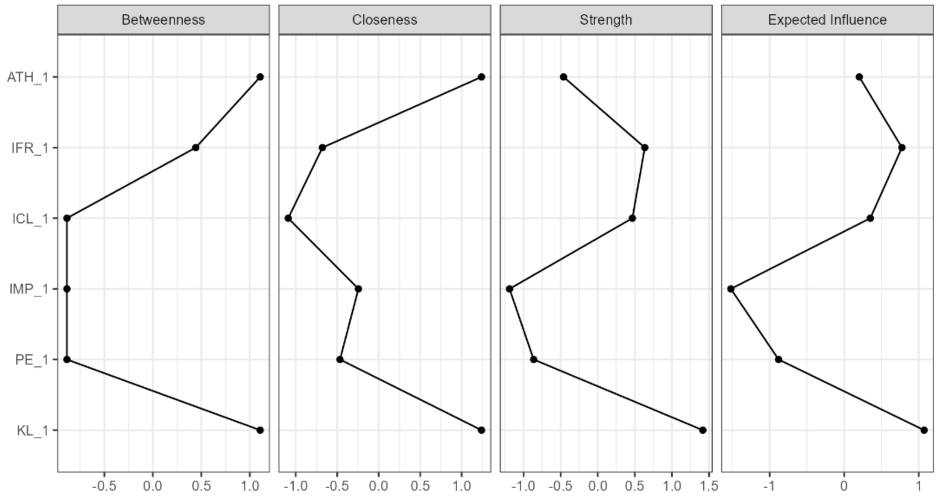

Bootstrap summary of Network

Type	Number of bootstraps
Nonparametric	1000

Edge Stability

Network

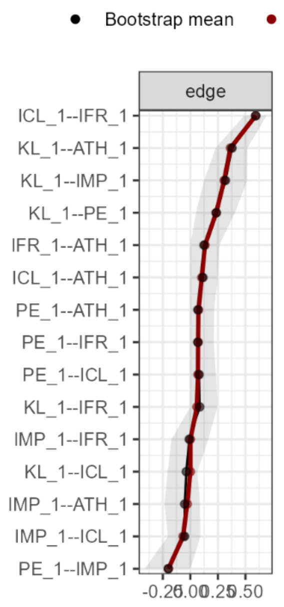

Centrality Stability

Network

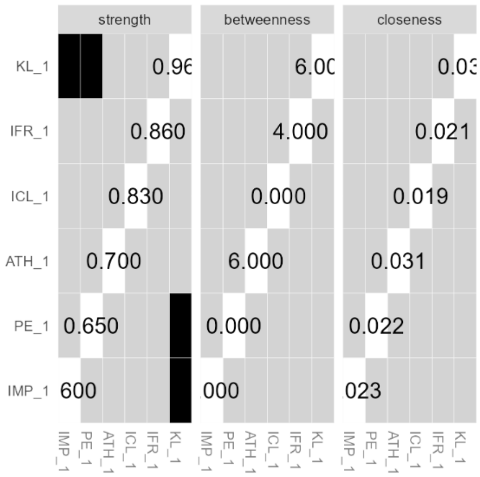

Network Analysis 2 ADHD

Summary of Network

Number of nodes	Number of non-zero edges	Sparsity
6	11 / 15	0.267

Centrality measures per variable

	Network			
**Variable**	**Betweenness**	**Closeness**	**Strength**	**Expected influence**
KL_2	1.826	1.353	0.559	0.750
PE_2	-0.913	-1.300	-1.127	-0.709
IMP_2	-0.913	-0.933	-1.327	-1.680
ICL_2	0.000	0.032	1.033	0.362
IFR_2	0.000	0.071	0.762	0.925
ATH_2	0.000	0.777	0.100	0.352

Weights matrix

	Network					
**Variable**	**KL_2**	**PE_2**	**IMP_2**	**ICL_2**	**IFR_2**	**ATH_2**
KL_2	0.000	0.167	0.123	0.108	0.102	0.271
PE_2	0.167	0.000	0.000	0.000	0.000	0.160
IMP_2	0.123	0.000	0.000	-0.121	0.030	0.000
ICL_2	0.108	0.000	-0.121	0.000	0.570	0.097
IFR_2	0.102	0.000	0.030	0.570	0.000	0.122
ATH_2	0.271	0.160	0.000	0.097	0.122	0.000

Network

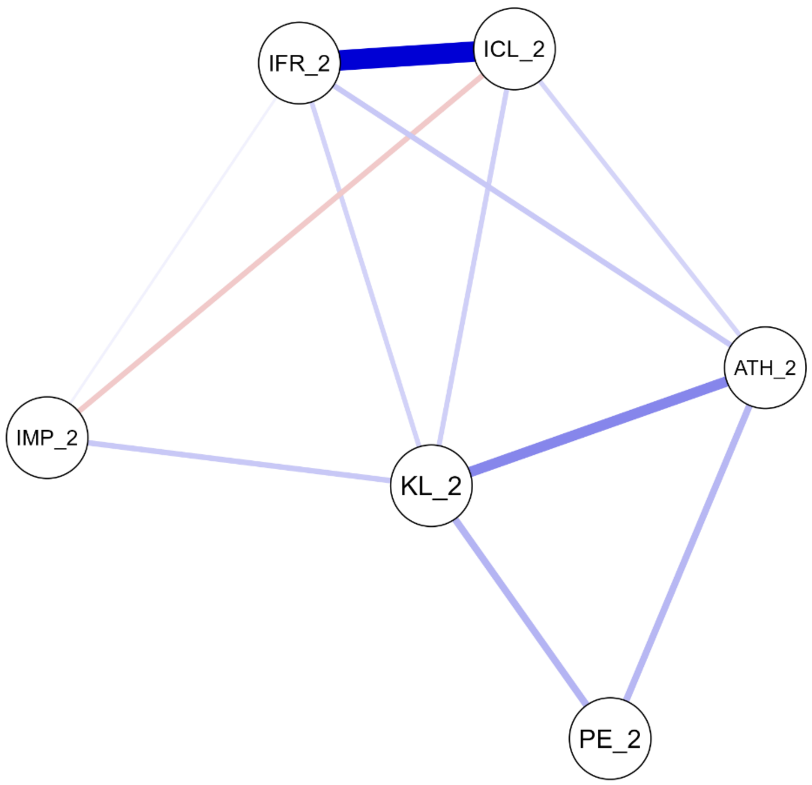

Centrality Plot

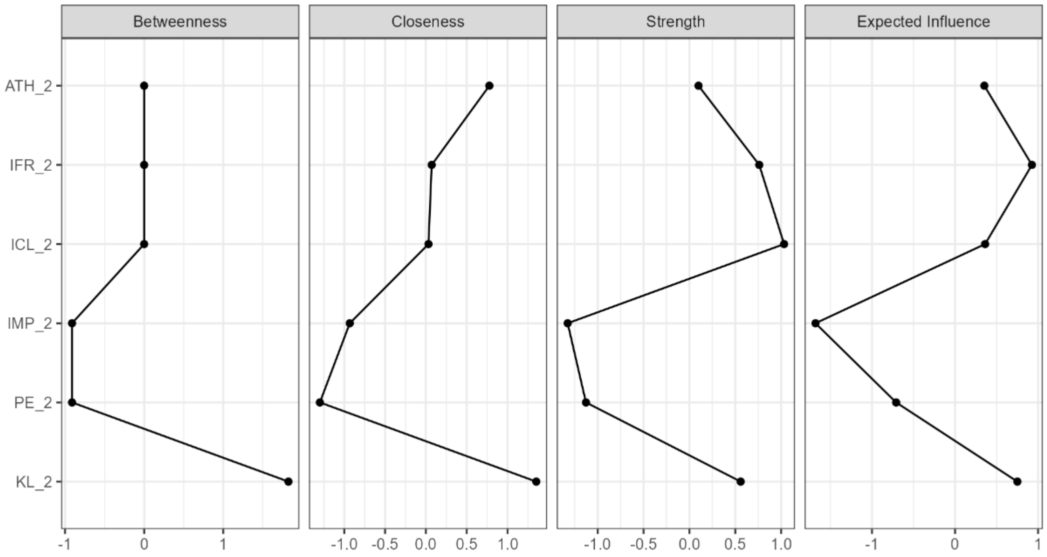

Bootstrap summary of Network

Type	Number of bootstraps
Nonparametric	1000

Edge Stability

Network

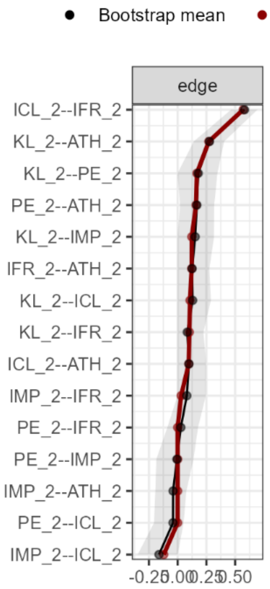

Centrality Stability

Network

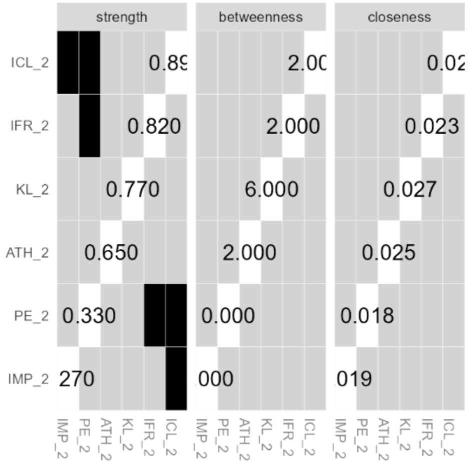

Network Analysis 3 Anxiety disorders

Summary of Network

Number of nodes	Number of non-zero edges	Sparsity
6	10 / 15	0.333

Centrality measures per variable

	Network			
Variable	Betweenness	Closeness	Strength	Expected influence
KL_3	1.523	1.022	0.746	0.746
PE_3	-0.190	0.420	-0.196	-0.196
IMP_3	-0.761	-1.712	-1.902	-1.902
ICL_3	0.952	0.029	0.769	0.769
IFR_3	-0.761	-0.519	0.403	0.403
ATH_3	-0.761	0.761	0.181	0.181

Weights matrix

	Network					
Variable	KL_3	PE_3	IMP_3	ICL_3	IFR_3	ATH_3
KL_3	0.000	0.332	0.083	0.128	0.071	0.210
PE_3	0.332	0.000	0.107	0.000	0.000	0.159
IMP_3	0.083	0.107	0.000	0.000	0.000	0.000
ICL_3	0.128	0.000	0.000	0.000	0.527	0.175
IFR_3	0.071	0.000	0.000	0.527	0.000	0.145
ATH_3	0.210	0.159	0.000	0.175	0.145	0.000

Network

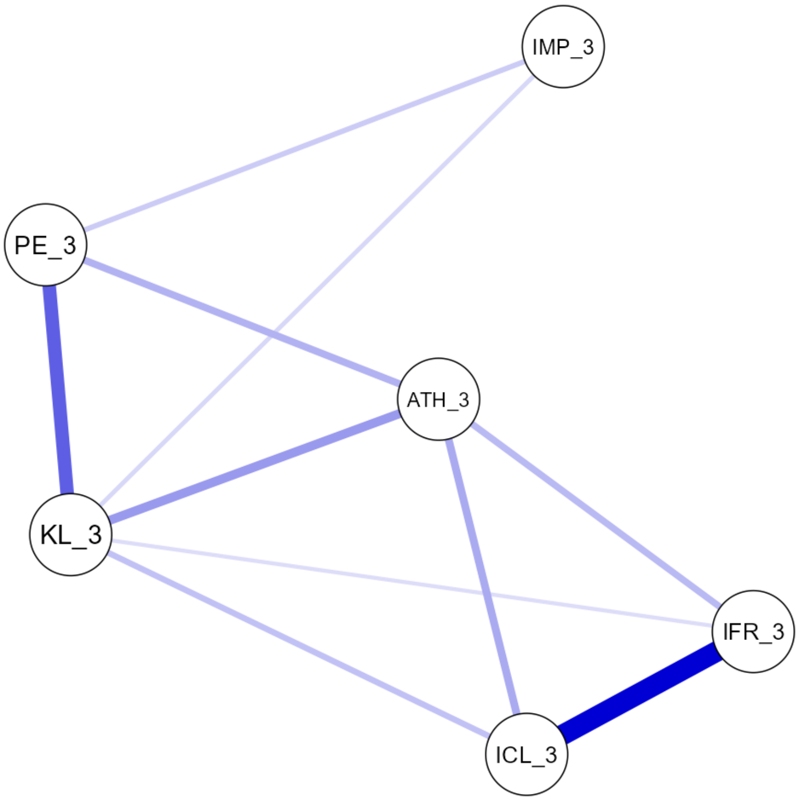

Centrality Plot

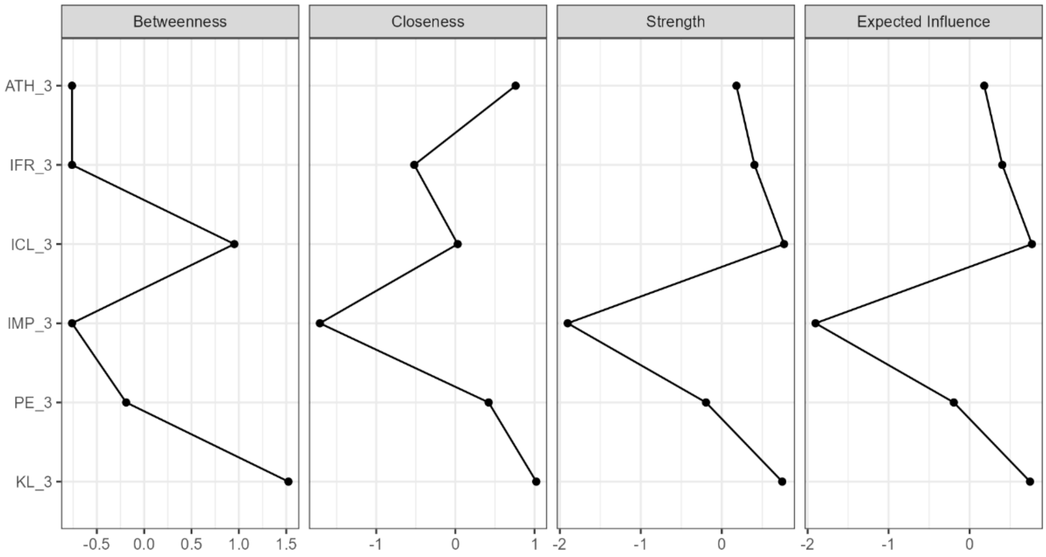

Bootstrap summary of Network

Type	Number of bootstraps
Nonparametric	1000

Edge Stability

Network

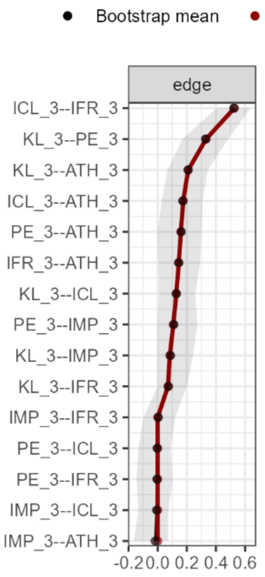

Centrality Stability

Network

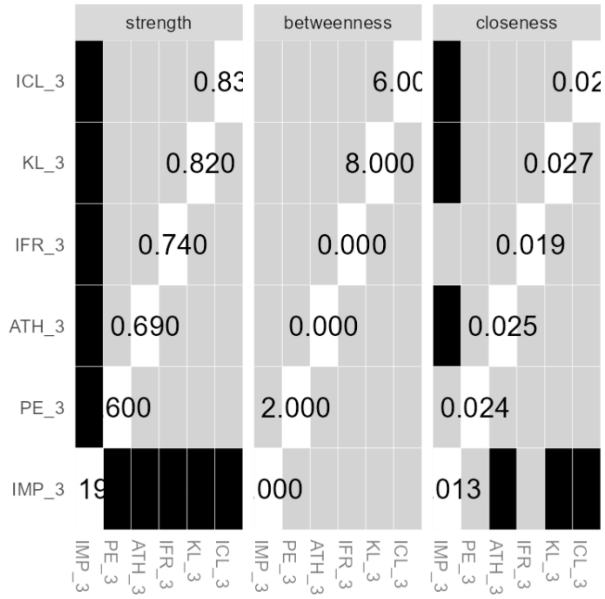

Network Analysis 4: Intellectual disability

Summary of Network

Number of nodes	Number of non-zero edges	Sparsity
6	11 / 15	0.267

Centrality measures per variable

	Network			
Variable	Betweenness	Closeness	Strength	Expected influence
KL_4	-0.408	-0.326	-0.168	-0.039
PE_4	-0.408	0.221	-0.275	-0.142
IMP_4	-0.408	-1.799	-1.628	-1.807
ICL_4	2.041	0.958	1.323	1.026
IFR_4	-0.408	0.822	0.702	0.796
ATH_4	-0.408	0.124	0.045	0.165

Weights matrix

	Network					
Variable	KL_4	PE_4	IMP_4	ICL_4	IFR_4	ATH_4
KL_4	0.000	0.152	0.000	0.087	0.098	0.139
PE_4	0.152	0.000	0.031	0.162	0.000	0.103
IMP_4	0.000	0.031	0.000	-0.051	0.000	0.000
ICL_4	0.087	0.162	-0.051	0.000	0.449	0.128
IFR_4	0.098	0.000	0.000	0.449	0.000	0.163
ATH_4	0.139	0.103	0.000	0.128	0.163	0.000

Network

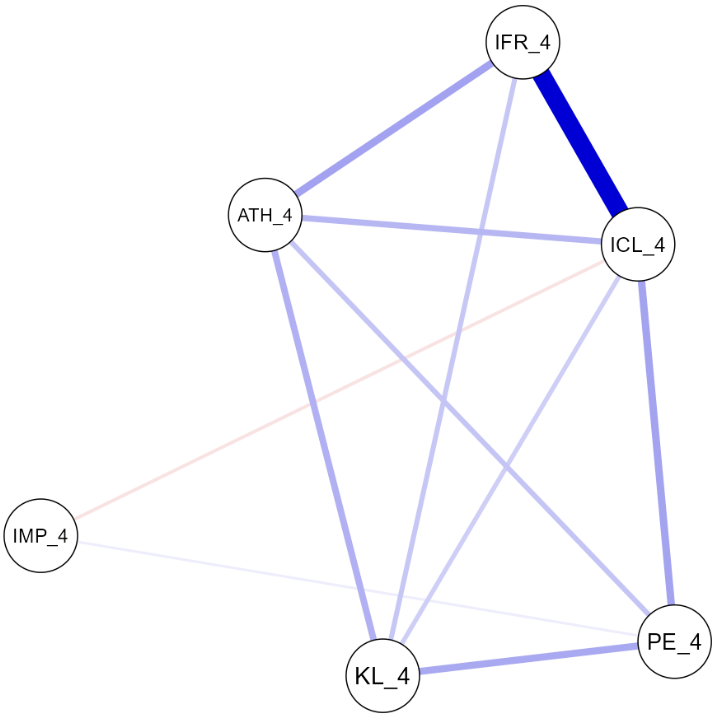

Centrality Plot

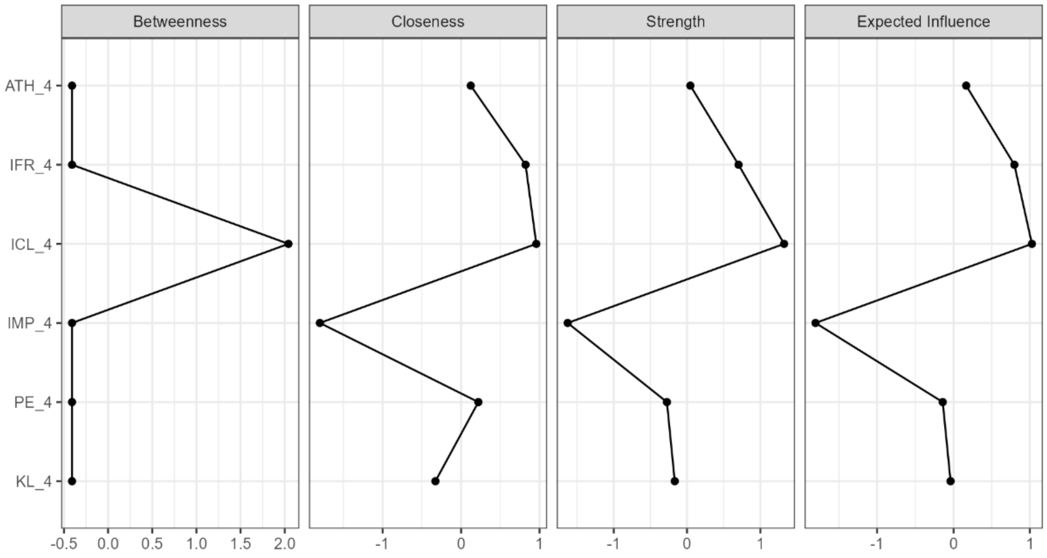

Bootstrap summary of Network

Type	Number of bootstraps
Nonparametric	1000

Edge Stability

Network

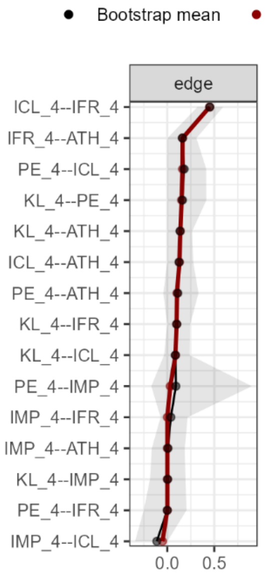

Centrality Stability

Network

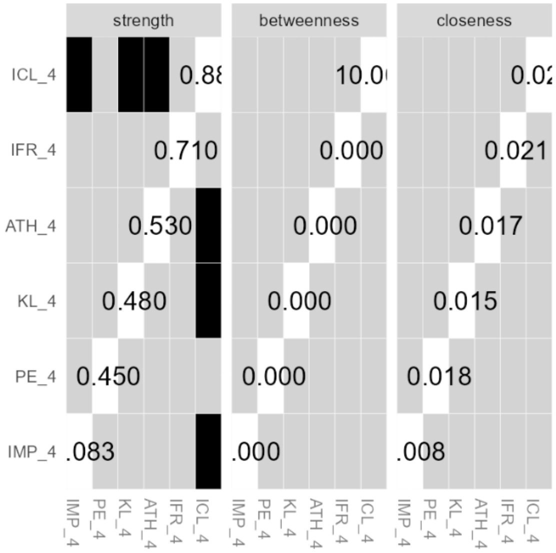

Network Analysis 5 Orthopaedic impairment

Summary of Network

Number of nodes	Number of non-zero edges	Sparsity
6	6 / 15	0.600

Centrality measures per variable

	Network			
Variable	Betweenness	Closeness	Strength	Expected influence
KL_5	0.716	0.000	-0.198	-0.198
PE_5	-0.895	0.000	-0.737	-0.737
IMP_5	-0.895	0.000	-1.231	-1.231
ICL_5	-0.895	0.000	0.842	0.842
IFR_5	0.716	0.000	1.474	1.474
ATH_5	1.253	0.000	-0.149	-0.149

Weights matrix

	Network					
Variable	KL_5	PE_5	IMP_5	ICL_5	IFR_5	ATH_5
KL_5	0.000	0.148	0.000	0.000	0.019	0.143
PE_5	0.148	0.000	0.000	0.000	0.000	0.000
IMP_5	0.000	0.000	0.000	0.000	0.000	0.000
ICL_5	0.000	0.000	0.000	0.000	0.615	0.005
IFR_5	0.019	0.000	0.000	0.615	0.000	0.176
ATH_5	0.143	0.000	0.000	0.005	0.176	0.000

Network

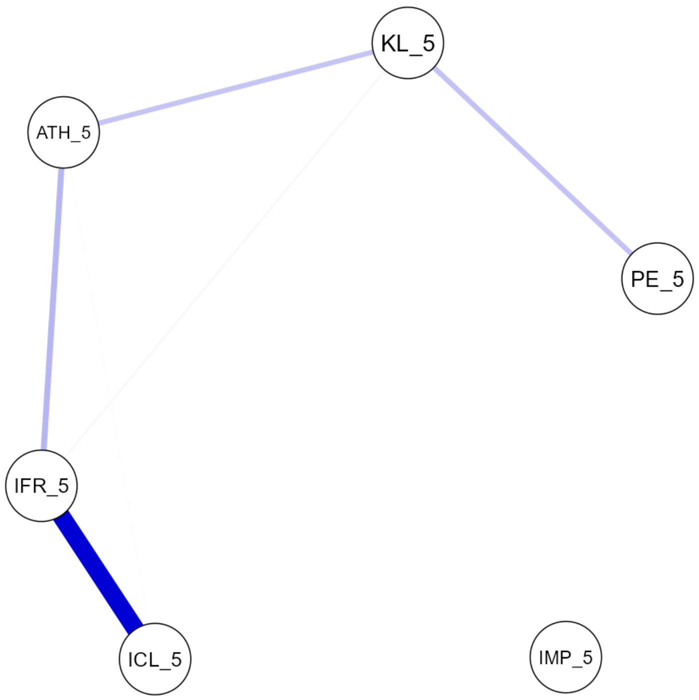

Centrality Plot

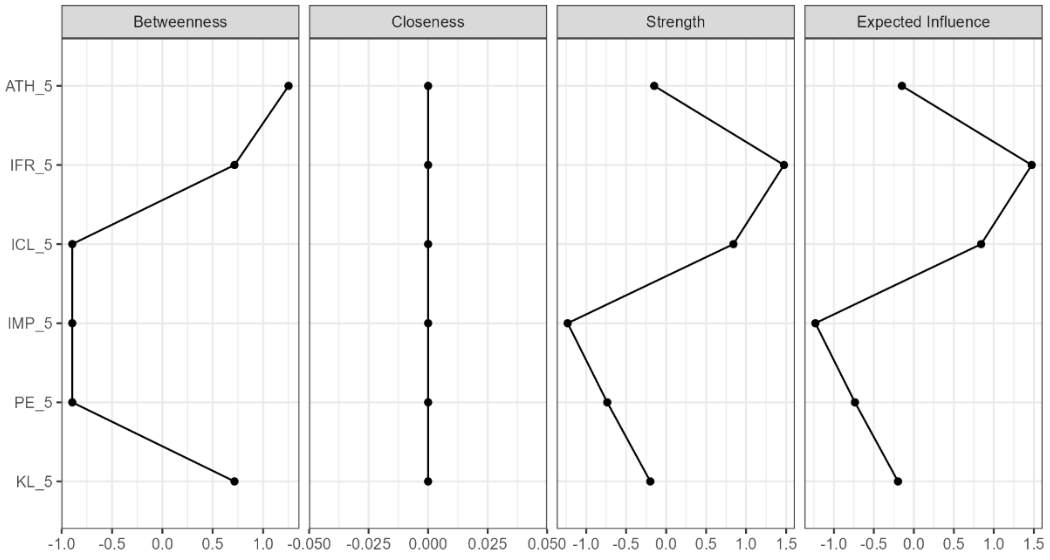

Bootstrap summary of Network

Type	Number of bootstraps
Nonparametric	1000

Edge Stability

Network

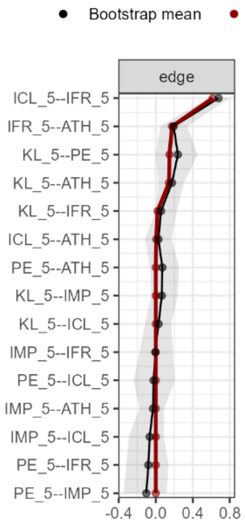

Centrality Stability

Network

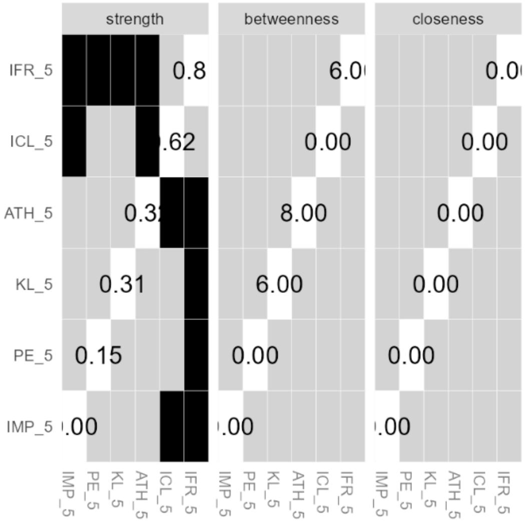

Network Analysis 8: Speech Impairment

Summary of Network

Number of nodes	Number of non-zero edges	Sparsity
6	12 / 15	0.200

Centrality measures per variable

	Network			
**Variable**	**Betweenness**	**Closeness**	**Strength**	**Expected influence**
KL_8	0.179	0.229	0.098	0.363
PE_8	-0.895	-0.373	-0.799	-0.310
IMP_8	-0.895	-1.736	-1.399	-1.626
ICL_8	-0.358	0.293	1.265	1.025
IFR_8	0.179	0.318	0.890	0.956
ATH_8	1.790	1.268	-0.055	-0.406

Weights matrix

	Network					
**Variable**	**KL_8**	**PE_8**	**IMP_8**	**ICL_8**	**IFR_8**	**ATH_8**
KL_8	0.000	0.203	0.048	0.093	0.068	0.186
PE_8	0.203	0.000	0.000	0.115	0.000	0.042
IMP_8	0.048	0.000	0.000	-0.038	0.000	-0.116
ICL_8	0.093	0.115	-0.038	0.000	0.593	0.068
IFR_8	0.068	0.000	0.000	0.593	0.000	0.147
ATH_8	0.186	0.042	-0.116	0.068	0.147	0.000

Network

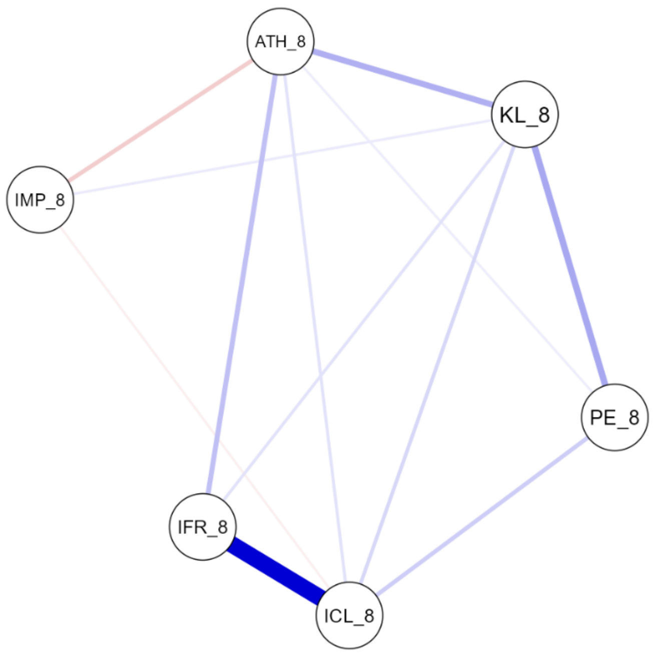

Centrality Plot

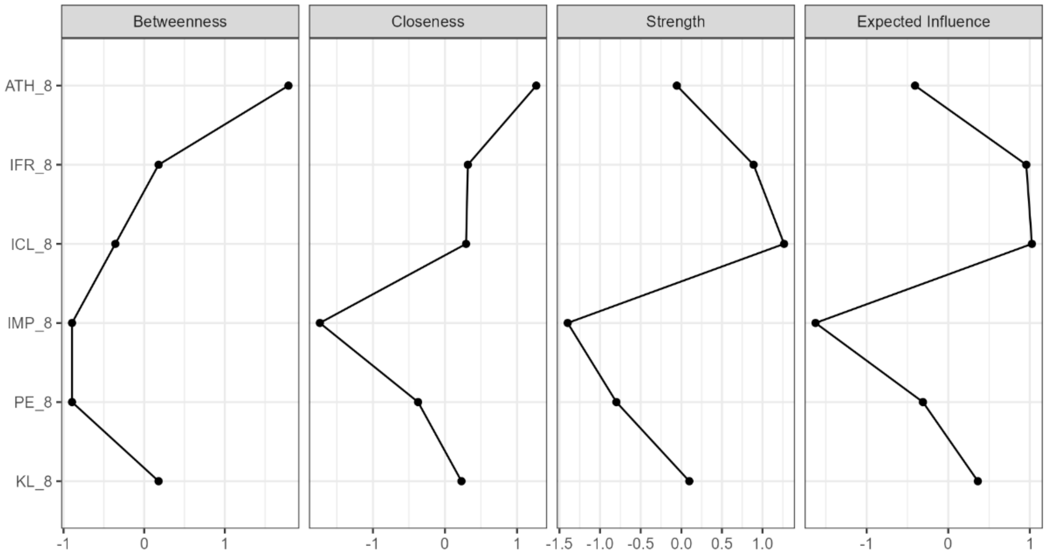

Bootstrap summary of Network

Type	Number of bootstraps
Nonparametric	1000

Edge Stability

Network

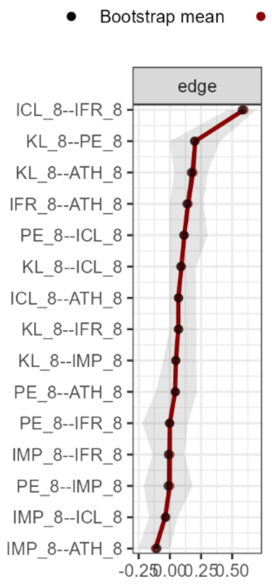

Centrality Stability

Network

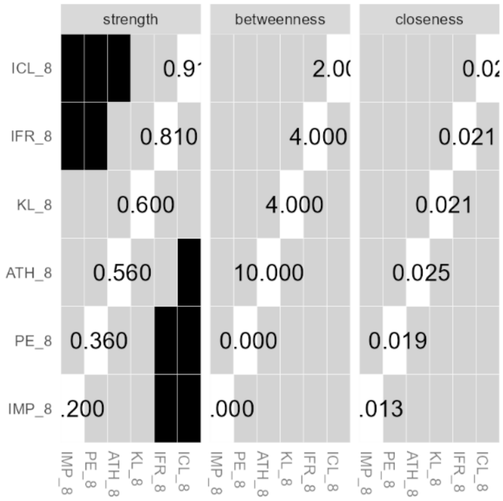

Supplement: Supplementary file 1 [file Supplementaryfile1.pdf]
